# Immunogenic radiation therapy for enhanced anti-tumor immunity *via* core-shell nanocomposite-mediated multiple strategies

**DOI:** 10.7150/thno.84500

**Published:** 2023-07-14

**Authors:** Naihan Huang, Along Qian, Yiming Zou, Miaoli Lin, Weilun Pan, Ming Chen, Wei Meng, Wenhua Zhang, Jinxiang Chen

**Affiliations:** 1 Guangdong Provincial Key Laboratory of New Drug Screening, Guangzhou Key Laboratory of Drug Research for Emerging Virus Prevention and Treatment, NMPA Key Laboratory for Research and Evaluation of Drug Metabolism, School of Pharmaceutical Sciences, Southern Medical University, Guangzhou, Guangdong 510515, China.; 2 College of Chemistry, Chemical Engineering and Materials Science, Soochow University, Suzhou 215123, China.

**Keywords:** radiation therapy, tumor microenvironment, anti-tumor immunity, immunogenic cell death

## Abstract

**Background:** Due to the immunosuppressive tumor microenvironment (TME), radiation therapy (RT)-mediated immune response is far from satisfactory. How to improve the efficacy of immunogenic RT by priming strong immunogenic cell death (ICD) is an interesting and urgent challenge.

**Methods:** A polyacrylic acid-coated core-shell UiO@Mn_3_O_4_ (denoted as UMP) nanocomposite is constructed for immunogenic RT *via* multiple strategies.

**Results:** Reshaping the TME *via* Mn_3_O_4_-mediated integration of O_2_ production, GSH depletion, ROS generation and cell cycle arrest, accompanied by Hf-based UiO-mediated radiation absorption, eventually amplifies UMP-mediated RT to induce intense ICD. With the potent ICD induction and reprogrammed tumor-associated macrophages, this synergetic strategy can promote dendritic cells maturation and CD8^+^ T cells infiltration, and potentiate anti-tumor immunity against primary, distant, and metastatic tumors.

**Conclusion:** This work is expected to shed light on the immunosuppressive TME-reshaping *via* multiple strategies to reinforce the immunogenic RT outcome and facilitate the development of effective cancer nanomedicine.

## Introduction

Radiation therapy (RT) is a widely used local therapy for cancer that utilizes high-energy radiation to ionize DNA or generate reactive oxygen species (ROS) for DNA damage in cancer cells 1. In theory, RT can activate a systemic immune response by inducing immunogenic cell death (ICD) to activate effector T cells 2, 3. Therefore, RT can in principle exert an anti-tumor effect on unirradiated distant tumors, which is called the abscopal effect 4. However, there are only 46 cases of RT-mediated abscopal effect reported from 1969 to 2014 with a median radiation dose of 31 Gy 5. Only several immunogenic tumors, including melanoma, renal cell carcinoma, and lymphoma, are mostly expected to have the abscopal effect during the treatment 6. Limited by weak radiation absorption and immunosuppressive tumor microenvironment (TME), RT alone can only induce a low level of ICD, thus providing insufficient antigens for anti-tumor immune activation 7. Therefore, how to improve the efficacy of radioimmunotherapy by priming strong ICD is an interesting and urgent challenge.

Tumors are soft tissues with low radiation absorption and deposition capacities, resulting in inadequate ROS generation. Therefore, in clinical practice, high radiation doses are often used to ensure the therapeutic effect 8. However, the generated ROS by applying high radiation is drastically eliminated due to the endogenous over-expressed glutathione (GSH), resulting in low cancer inhibitory potency 9. Most importantly, the high radiation dose application will exacerbate the degree of hypoxia in tumors, resulting in an insufficient reaction between RT-generated DNA radicals with molecular oxygen. Hypoxia thus halted further damage to the DNA, leading to radioresistance. In addition, hypoxia also upregulates hypoxia-inducible factor-1α (HIF-1α), which further promotes radioresistance and tumor metastasis 10.

A variety of nanomaterials have been studied and applied in the enhancement of intracellular radiation deposition 11. Among them, metal-organic frameworks (MOFs), formed by coordination interactions between metal centers and organic ligands, have attracted great attention due to their unique advantages 12. The metal centers containing high-Z elements (*e.g.*, Gd, Hf, and Bi) can serve as radiosensitizers because of their strong X-ray attenuation ability. In particular, hafnium as a metal center has some advantages, such as high coordination ability and biocompatibility (*e.g*., hafnium oxide nanoparticle NBTXR3 has been used in clinical RT 13). Besides, the periodic structures of MOFs allow the effective use of secondary photons and electrons from the enhanced photoelectric and/or Compton effects. Moreover, the porous structure facilitates fast diffusion of ROS to attack tumor cells during RT.

On the other hand, tumor-associated macrophages (TAMs), as influenced by the TME, are mostly M2 phenotype. As the major tumor-infiltrating immunosuppressive cells, these M2-like macrophages play an important role in tumor progression and metastasis 14, 15. M2-like macrophages occupy a considerable area of tumor tissues, especially in breast cancer (>50%) 16. Fortunately, macrophages have strong plasticity and can be repolarized into tumoricidal M1 phenotype 17, 18.

**Scheme 1:**
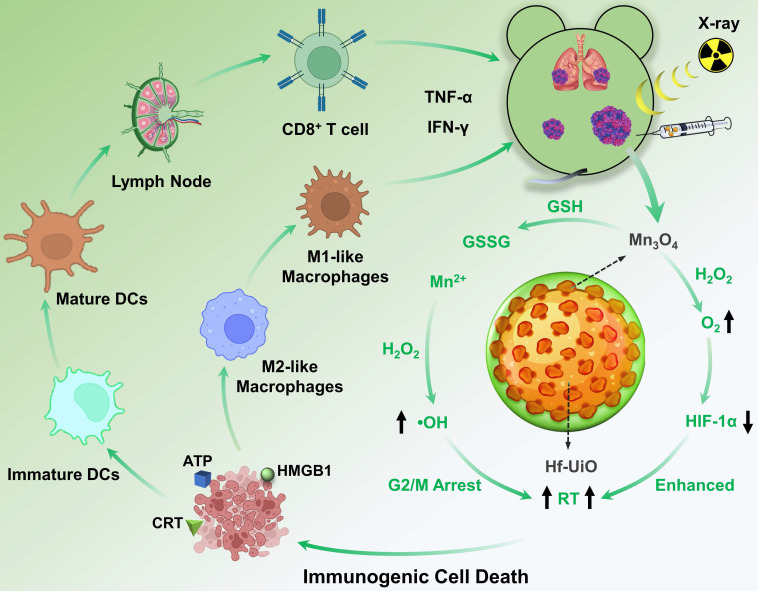
Schematic illustration of UMP-mediated multiple strategies for immunogenic radiation therapy

To achieve positive modulation of the immunosuppressive TME and radiosensitization for an advanced RT and immunogenicity, we designed and constructed a core-shell structure of UiO@Mn_3_O_4_ (UM) by coating Mn_3_O_4_ particles on UiO-66(Hf)-NH_2_ (UiO, originally reported by Lillerud's group in the University of Oslo 19). After polyacrylic acid (PAA) coating, the obtained UiO@Mn_3_O_4_@PAA (UMP) could serve as an efficient nanoregulator for alleviating hypoxia, and inducing oxidative stress *via* GSH depletion-enhanced Fenton-like reaction. Therefore, UMP re-assorted cell cycle distribution (*i.e.*, at the gap 2/mitosis (G2/M) phases), consequently contributing to enhanced tumoral radiosensitivity. Upon 4 Gy dose irradiation, Hf-based UiO could serve as a radiosensitizer to give substantial amounts of ROS. Combined with Mn_3_O_4_ mediated-TME modulation, this synergistic effect induced a workable ICD, resulting in reprogramming of M2-like macrophages to M1-like, maturation of dendritic cells (DCs), activation of CD8^+^ T cells, secretion of inflammatory cytokines TNF-α and IFN-γ, and ultimately initiation of a powerful anti-tumor immune response. Thus, UMP demonstrated satisfactory efficiency in eliminating primary, distant, and metastatic tumors (**Scheme 1**).

## Methods

### Materials and measurements

All chemical reagents were used directly without further purification. HfCl_4_ (99.99%) and Mn(OAc)_2_∙4H_2_O were purchased from Shanghai Maclean Biochemical Technology Co., Ltd. Methylene blue (MB) and 5, 5-dimethyl-l-pyrroline-N-oxide (DMPO) were purchased from Shanghai Aladdin Biochemical Technology Co., Ltd. Ferrostatin-1 (Fer-1) and H_2_O_2_ (30%) was purchased from Yike Biological Technology Co., LTD (Guangzhou, China). C11-BODIPY^581/591^ was bought from Invitrogen Corp. 5, 5′-Dithiobis-(2-nitrobenzoic acid) (DTNB) and methylthiazoletatrezolium (MTT) were purchased from Beijing Coolaibo Technology Co., Ltd. The ROS detection kit 2′,7′-dichlorofluorescein diacetate (DCFH-DA), Calcein-AM/PI kit, ATP assay kit, TNF-α ELISA Kit, IFN-γ ELISA Kit, tetramethylrhodamine ethyl ester (TMRE) and 4',6-diamidino-2-phenylindole (DAPI) were purchased from Beyotime Biotechnology Co., Ltd (Shanghai, China). Intracellular GSH detection reagent was obtained from J&K Chemical Co., LTD (Shanghai, China). All reagents relevant to cell culture were obtained from GIBCO Invitrogen Corp. FITC Anti-Mouse CD11c Antibody [N418], APC Anti-Mouse CD86 Antibody [GL-1], APC Anti-Mouse CD206 Antibody [C068C2], and PE Anti-Mouse CD80 Antibody [16-10A1] were purchased from Elabscience Biotechnology Co., Ltd.

The UV-Vis absorption spectroscopy was conducted by Perkin Elmer LS55 UV/Vis spectrometer. The transmission electron microscopy (TEM) was performed on JEOL JEM 100CXII operated at 200 kV. Scanning electron microscopy (SEM) was conducted by EMAXevolutin X-Max80/EX-270. The dynamic light scattering (DLS) and surface charge (zeta potential) of nanoparticles were conducted by Zetasizer Nano-ZS ZEN3600 (Malvern Instruments, UK). The powder X-ray diffraction (PXRD) patterns were recorded on a Bruker D8 GADDS (General Area Detector Diffraction System) micro-diffractometer equipped with a VANTEC-2000 area detector with Φ rotation. The metal ions concentration was measured by inductively coupled plasma-mass spectrometry (ICP-MS) taken on an ICAP 6300 of Thermo Scientific. The dissolved oxygen measurement was conducted by JPB-607A. The fluorescence microscopy images were observed on a confocal laser scanning microscopy (CLSM, Nikon C1-Si TE2000, Japan). The flow cytometry was performed using a BD FACS Caliber instrument (BD Biosciences, San Jose, USA). The cytotoxicity assay was carried out on a multifunction microporous plate detector (Infinite M1000 Pro, Tecan, Switzerland). Magnetic resonance imaging (MRI) was performed on a small animal magnetic resonance imaging system (PharmaScan70/16 US). Electron paramagnetic resonance (EPR, A300, Bruker) was used to detect •OH generation.

### Preparation of UiO-66(Hf)-NH_2_ (UiO), UiO@Mn_3_O_4_ (UM), and UiO@Mn_3_O_4_@PAA (UMP)

The modulated hydrothermal synthesis of UiO was based on the modification of a reported procedure 20. In a typical process, 2-aminoterephthalic acid (1.81 g, 10 mmol) and HfCl_4_ (3.204 g, 10 mmol) were co-dissolved in a mixture of 100 mL water/acetic acid (2/3, 3/2, 3.5/1.5, 4/1, 4.5/0.5, v/v, mL) in a flask. After sonicating for 10 min, the flask was then placed in a 100 ºC oil bath immediately and refluxed for 24 h to yield colorless powder. The obtained powder was washed with methanol twice and soaked in methanol for another 3 days at room temperature, during which time the product was centrifuged every day to remove the supernatant.

To synthesize UM with different morphologies, the powder of Mn(OAc)_2_·4H_2_O and UiO with different mass ratio of 1:1 (0.7 g/0.7 g), 4:1 (2.8 g/0.7 g), 8:1 (5.6 g/0.7 g) were co-dispersed in 300 mL DMF and loaded into a 500 mL round-bottomed flask. After sonicating for 10 min, the mixture was heated to 120 ºC and reacted for 8 h under stirring. The brown products were collected by centrifugation and washed several times with ethanol. Finally, the purified UM was dried in an oven at 80 ºC for 24 h. To synthesize UMP, the powder of UM (1.0 g) and PAA (5.0 g) were co-dispersed in a mixture of 200 mL water/ethanol (1/1, v/v) in a flask. After sonicating for 10 min, the mixture was stirred for 10 h and collected by centrifugation, followed by washing with water.

### GSH-mediated Mn^2+^ release from UM

UM (200 μg/mL) was pretreated with different concentrations of GSH (0, 0.1, 0.5, 1, 10 mM) for 15 min in water. The supernatant was collected by centrifugation at 20000 rpm for 10 min and the Mn^2+^ content in the supernatant was measured by ICP-MS.

### Time-dependent degradation profiles of GSH-mediated UM

Under dark conditions, UM (50 μg/mL) was mixed with 1 mM GSH at a different time in water. The absorbance of the solution was recorded at the indicated time points by the UV-Vis absorption spectra.

### GSH-dependent degradation profiles of UM

Under dark conditions, UM (50 μg/mL) was mixed with different concentrations of GSH (0, 0.4, 0.8, 1.5, 2.5 mM) for 15 min in water. The absorbance of the solution was recorded by the UV-Vis absorption spectra.

### GSH depletion capacity of UM

The GSH amount was detected by Ellman's reagent. Under dark conditions, different concentrations of UM (0, 25, 50, 75, 100, 125, 150, 175 μg/mL) and GSH (1 mM) mixture reacted for 12 h. After centrifugation (20000 rpm, 10 min), DTNB (0.05 mM) and PBS solution (10 mM, pH = 7.4) were added to the supernatant. Then, the mixture was incubated at 37 ºC for 30 min, and the absorbance change of DTNB at 250-600 nm was measured.

### Fenton-like •OH production of UM with GSH addition

The ability of UM to catalyze the production of •OH in the presence of GSH was confirmed by the degradation of methylene blue (MB) under dark conditions. Briefly, 25 mM NaHCO_3_ solution containing UM (50 μg/mL) and different concentrations of GSH (0, 1, 2, 3, 4, 5, 6, 7, 8, 9, 10 mM) was shaken at 37 ºC for 15 min. After centrifugation (20000 rpm, 10 min), 6 μg/mL MB and 10 mM H_2_O_2_ were added to the supernatant. Then, the mixture was incubated at 37 ºC for 30 min, and the absorbance change of MB at 500-800 nm was measured.

The •OH generation ability of UM was also evaluated by the EPR measurement. Briefly, 25 mM NaHCO_3_ solution containing UM (50 μg/mL) and different concentrations of GSH (0 and 3 mM) was shaken at 37 ºC for 15 min. After centrifugation (20000 rpm, 10 min), DMPO was added into the supernatant and detected immediately after addition of H_2_O_2_. DMPO was used as control.

### Cell culture

4T1 and L929 cells were cultured in an RPMI-1640 medium containing 10% fetal bovine serum and 1% antibiotics (penicillin-streptomycin, 10000 U/mL). RAW 264.7 cells were cultured in a DMEM medium containing 10% fetal bovine serum and 1% antibiotics.

### Cellular uptake behaviors of UMP

The 35 mm CLSM dish-adhered 4T1 cells were incubated with IR780-labeled UMP (50 µg/mL) for 2 h, 4 h, and 6 h. Then, these cells were rinsed by PBS and imaged by CLSM to investigate the uptake of UMP (Ex: 640 nm, Em: 700 nm). Besides, the Hf and Mn content of UMP-treated 4T1 cells at different times were confirmed by ICP-MS.

### Intracellular oxygen and HIF-1α assay

Under hypoxic conditions (5% CO_2_/1% O_2_), 4T1 cells were seeded into 35 mm CLSM-exclusive culture dishes for 24 h. For oxygen evaluation, 30 μM of [Ru(dpp)_3_]Cl_2_ was added for 4 h. After that, they were incubated with 0.1 mM H_2_O_2_ and UiO (50 µg/mL) or UMP (50 µg/mL) for 6 h. Then, the cells were rinsed with PBS three times and imaged *via* CLSM (Ex: 488 nm, Em: 615 nm). For immunofluorescence staining of HIF-1α, the cells were incubated with UiO (50 µg/mL) or UMP (50 µg/mL) for 6 h. Then, the cells were further treated with 4% paraformaldehyde and 0.2% Triton X-100 and then incubated with HIF-1α rabbit monoclonal antibody (dilution 1:1000) overnight at 4 ºC. After washing with PBS five times, the cells were incubated with a secondary antibody (CoraLite594-conjugated Goat Anti-Mouse IgG (H+L), Proteintech, SA00013-3, China) at a 1/500 dilution for 2 h at room temperature in the dark. The nuclei (blue) were stained with DAPI for 20 min at room temperature. After washing with PBS five times, the cells were imaged by CLSM.

### MTT assays

The cells were seeded in 96-well plates and incubated for 24 h. Then the original medium was replaced with another 100 µL of fresh medium containing a series of concentrations of UiO or UMP. There were 6 wells in each group. After incubation for 6 h, the medium was replaced by 100 µL of fresh medium. For the RT group, the cells were irradiated with a 4 Gy X-ray. After that, the cells were cultured for 24 h. Then the medium was discarded, followed by the addition of 100 μL of MTT (0.5 mg/mL)-containing medium and the incubation continued for 4 h. After discarding the medium, 100 µL of DMSO was added to each well and shaken for 10 min on a shaker, and the absorbance at 490 nm was measured using a microplate reader (Infinite M1000 Pro, Tecan, Switzerland).

### Living/dead cell staining experiment

4T1 cells were seeded into the 6-well plates and incubated for 24 h and then treated with UiO (100 µg/mL) or UMP (100 µg/mL). After incubation for 6 h, the medium was replaced by 2 mL of fresh medium. For the RT group, the cells were irradiated with a 4 Gy X-ray. After that, the cells were cultured for 6 h. The cells were stained with Calcein-AM (8 µM) and PI solutions (2 µM) and incubated for 30 min in the dark condition. Lastly, the cells were observed by CLSM (green fluorescence indicates live cells and red fluorescence indicates dead cells).

### Intracellular GSH depletion

4T1 cells were cultured in the 35 mm CLSM-exclusive culture dishes for 24 h and then incubated with UMP (50 μg/mL) for 6 h. Then, the cells were washed with PBS and stained with GSH detection probe Na-8 (10 μM) for 30 min. The cells were washed thoroughly before being observed under CLSM (Ex: 405 nm, Em: 498 nm).

Six-well plate-adhered 4T1 cells were incubated for 24 h before use. Different concentrations of UMP (0, 50, and 100 μg/mL) were added and cultured for 6 h. Then the cells were washed with PBS thrice and lysed with 2% Triton-X-100 solution (Prepared with PBS buffer, 10 mM, pH = 7.4). After centrifugation (20000 rpm, 10 min), DTNB was added to the supernatant. Afterward, the mixture was incubated at 37 ºC for 30 min, and the absorbance change of TNB at 412  nm was measured. The percentage content of GSH was acquired based on the comparison with the GSH content of untreated cells.

### Intracellular ROS assay

Cells were seeded into 35 mm CLSM-exclusive culture dishes for 24 h. After that, they were incubated with UiO (50 µg/mL) or UMP (50 µg/mL) for 6 h. For ROS evaluation, the culture medium was replaced by DCFH-DA solution (10 μM) for 20 min and the cells were irradiated with or without a 4 Gy X-ray. The fluorescence was observed by CLSM.

### Intracellular lipid peroxide (LPO) assay

Cells were seeded into 35 mm CLSM-exclusive culture dishes for 24 h. 10 mM GSH or Fer-1 (ferroptosis inhibitors) was added in the cells before incubating with UMP (50 µg/mL). After 6 h, cells were incubated with C11-BODIPY^581/591^ fluorescent probe (10 μM) for 30 min. The fluorescence was observed by CLSM.

### Mitochondria membrane potential

4T1 cells were seeded into 35 mm CLSM-exclusive culture dishes for 24 h. After that, they were incubated with UMP (50 µg/mL) for 6 h. Then the medium was discarded, followed by the addition of 20 nM TMRE for 30 min. The cells were washed thoroughly before being observed under CLSM (Ex: 514 nm, Em: 575 nm).

### Cell cycle analysis

Six-well plate-adhered 4T1 cells were incubated with UMP (10 μg/mL) for 24 h. After incubation, those cells were collected by centrifugation (1500 rpm, 2 min), washed with PBS, and then incubated with propidium iodide (PI, 50 µg/mL) and RNase A for 30 min. The flow cytometry assay was employed for cell cycle analysis.

### Intracellular immunofluorescence staining of γ-H2A.x

Under hypoxic conditions (5% CO_2_/1% O_2_), 4T1 cells were seeded into 35 mm CLSM-exclusive culture dishes for 24 h. After that, they were incubated with UiO (50 µg/mL) or UMP (50 µg/mL). After incubation for 6 h, the medium was replaced by 1 mL of fresh medium. For the RT group, the cells were irradiated with X-ray at the dose of 4 Gy. After that, the cells were cultured for 6 h. Then the medium was discarded, followed by the addition of 4% paraformaldehyde and 0.2% Triton X-100 and then incubated with γ-H2A.x rabbit monoclonal antibody (dilution 1:500) overnight at 4 ºC. After washing with PBS five times, the cells were incubated with a secondary antibody at a 1/500 dilution for 2 h at room temperature in the dark. The nuclei (blue) were stained with DAPI for 20 min at room temperature. After washing with PBS five times, the cells were imaged by CLSM.

### Immunogenic cell death

4T1 cells were seeded into 35 mm CLSM-exclusive culture dishes for 24 h and incubated with UiO (50 µg/mL) or UMP (50 µg/mL) for 6 h. Then the medium was replaced by 1 mL of fresh medium. For the RT group, the cells were irradiated with a 4 Gy X-ray. After 4 h, the concentration of ATP in the supernatant was measured using an ATP assay kit, according to the manufacturer's instructions. After further culturing for 20 h, the cell surface expression levels of CRT were observed by their immunofluorescence *via* CLSM. In brief, cells were fixed in 4% paraformaldehyde for 10 min and stained with rabbit anti-CRT antibody overnight at 4 ºC. After washing with PBS five times, the cells were stained with Alexa Fluor® 594-conjugated secondary anti-rabbit antibody for 2 h at room temperature. After washing with PBS five times, the cells were stained with DAPI for 20 min and imaged by CLSM. Similarly, rabbit anti-HMGB1 antibody was used to detect HMGB1 after permeabilization of the cells with 0.2% Triton X-100.

### TAMs reprogramming

RAW 264.7 cells were seeded into 35 mm CLSM-exclusive culture dishes for 24 h and stimulated with 20 ng/mL interleukin-4 (IL-4) for 24 h to polarize them into M2-like macrophages. Then the cells were washed thrice and incubated with UMP + RT-treated 4T1 cells culture medium for another 24 h. Final analysis using immunofluorescence and flow cytometry.

### Hemolysis tests

The fresh mouse blood was washed three times with PBS. This is followed by mixing the obtained red blood cell suspension with 1% Triton X-100 (positive group), PBS (negative group), and various concentrations of UMP for 8 h, respectively.

### T_1_-weighted magnetic resonance imaging

Briefly, different concentrations of UMP (0, 0.1, 0.25, 0.5, 0.75, and 1 mM Mn concentrations) and 10 mM GSH were shaken at 37 ºC for 15 min under dark conditions. After centrifugation (20000 rpm, 10 min), the supernatant was transferred to 2 mL tubes. The curve fitting of 1/T_1_ relaxation time (s^-1^) versus the Mn^2+^ concentration achieved the r_1_ relaxivity values. For *in vivo* MRI, UMP was administered to the 4T1 tumor-bearing mice by intratumoral injection on the right side. MR scan images were taken at 24 h after UMP administration.

### *In vivo* anticancer studies

Female BALB/c mice (4 weeks old) were obtained from Southern Medical University Laboratory Animal Center and all animal experiments were conducted following the guidelines of the Regional Animal Laboratory Ethics Committee and the Southern Medical University Animal Care Use Committee (Permit Number: 44002100031030). The tumor volume was calculated as V = L*W^2^/2, where L and W refer to the length and width of the tumor, respectively.

The bilateral 4T1 breast tumor model was established by subcutaneous injection of 4T1 cells (1.5 × 10^6^) in the right hind limb (primary) and left hind limb (distant tumor). When the primary tumor volume reached about 100 mm^3^, mice were divided into 6 groups randomly and intratumorally injected with PBS, UiO, and UMP (50 mg/kg), respectively. After 24 h, the mice were irradiated with a dose of 4 Gy for primary tumors or remained with no additional treatment. On day 3, one mouse was sacrificed for each group, and the tumors were collected for staining. For analyzing DCs maturation in the tumor-draining lymph nodes (TDLNs), cells were stained by FITC Anti-Mouse CD11c Antibody, APC Anti-Mouse CD86 Antibody, and PE Anti-Mouse CD80 Antibody. Flow cytometric data acquisition was performed with BD LSRFortessa X-20, and the data were processed using FlowJo software. Serum samples were isolated from mice for analysis. Tumor necrosis factor-α (TNF-α) and interferon-gamma (IFN-γ) were analyzed with ELISA kits according to the vendor's instructions. The body weight and tumor volumes for the remaining mice were recorded every two days. After 16 days of observation, the mice were sacrificed and the tumors were collected for photography and weight recording. We also determined the tumor growth inhibition (TGI) according to the equation TGI (%) = [(Wc - Wt)/Wc] × 100%, in which Wc and Wt respectively represent the mean weight of control tumors and treated tumors. The main organs (heart, liver, spleen, lung, and kidney) were collected for H&E staining.

To establish the lung metastatic model, six days after mice were inoculated with 4T1 cells (1.5 × 10^6^) on the right posterior side back of the mice (the primary tumor), the second batch of 4T1 cells (2 × 10^5^) were induced by tail vein injection 21-23. After 24 h, the primary tumor was treated with PBS, UiO, and UMP (50 mg/kg), respectively. After 24 h, the mice were irradiated with a dose of 4 Gy for primary tumors or remained with no additional treatment. On day 16, mice were sacrificed and the lungs were fixed in Bouin's solution and collected for H&E staining. All the above staining procedures *in vivo* were carried out by Servicebio Biotechnology Co., Ltd., Wuhan.

## Results and discussion

### Preparation and characterization of UMP

**Figure 1:**
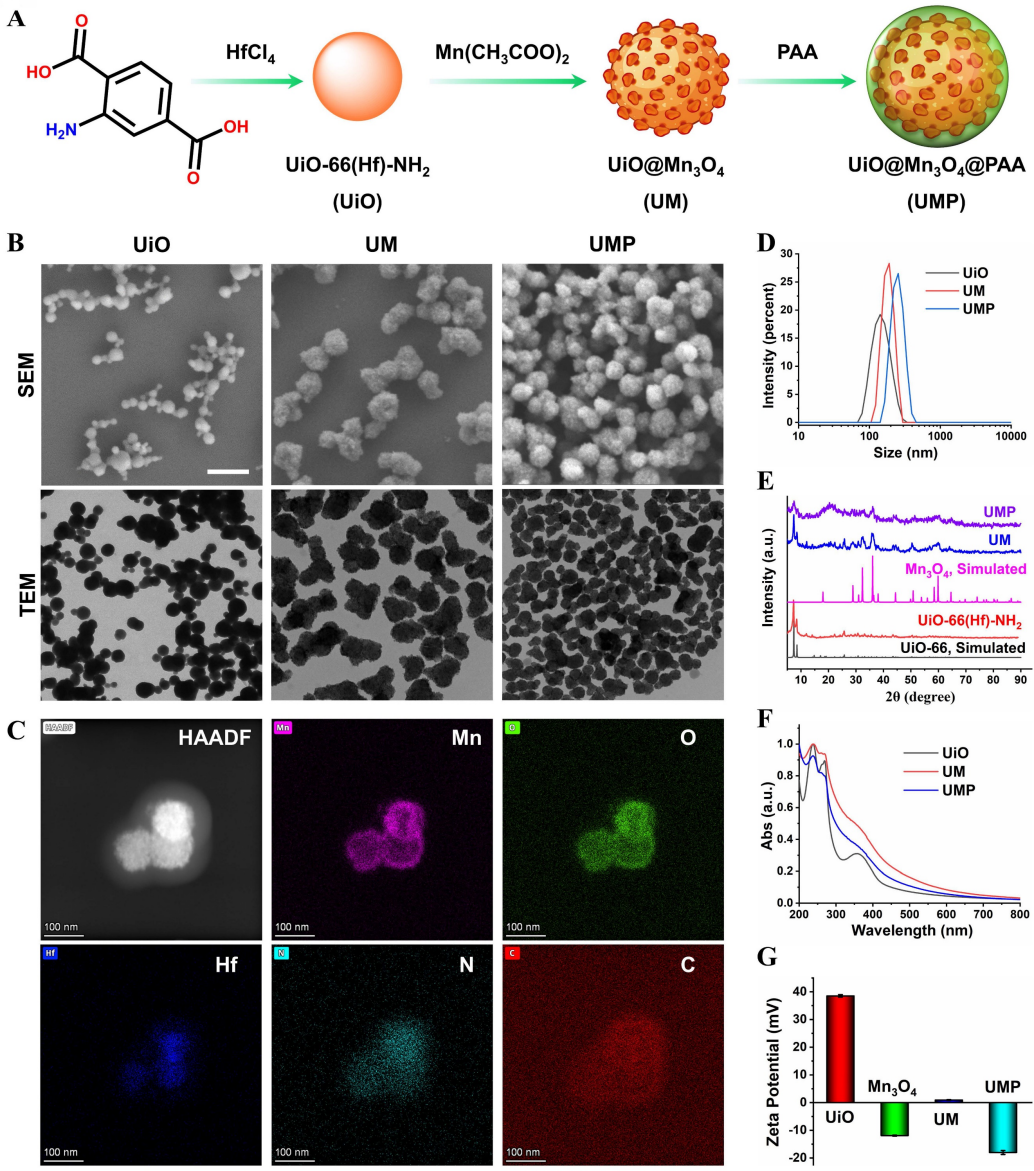
(A) Schematic illustration of UMP synthesis. (B) The SEM and TEM images of UiO, UM, and UMP. (C) The STEM-HAADF image and corresponding EDS elemental mapping of Mn, O, Hf, N, and C of UMP. The size distribution (D), the PXRD analysis (E), the UV-Vis absorption spectra (F), and the zeta potential (G) of various nanoparticles.

The synthesis process of UMP is shown in the **Figure 1A**. To construct UM, UiO was first prepared *via* a modulated hydrothermal method. A series of UiO with different particle sizes could be readily obtained by adjusting the ratio of water and acetic acid (**Table S1**). Scanning electron microscopy (SEM) and transmission electron microscopy (TEM) showed that the as-synthesized UiO nanoparticles (water/acetic acid, 80/20 mL) with the spherical structure were well dispersed in ethanol (**Figure 1B**) and its average diameter was 144.4 nm *via* dynamic light scattering (DLS) measurement (**Figure 1D**). The core-shell UM was then obtained by growing Mn_3_O_4_ nanoparticles (**Figure** S**1**) on the UiO surface. As shown in **Figure S2**, a series of UM with different morphologies could be easily obtained in the gram scale by adjusting the mass ratio of Mn(OAc)_2_·4H_2_O and UiO. Both SEM and TEM showed that the core-shell UM nanoparticles formed by the mass ratio between Mn(OAc)_2_·4H_2_O and UiO being 4 : 1 were well dispersed in ethanol with its mean DLS particle size being 182.5 nm. Additionally, the polymer dispersity index (PDI) value of UiO and UM was 0.038 and 0.073, indicating that such nanoparticles exhibited good stability and dispersibility in ethanol. Unfortunately, UM has poor dispersibility in water due to the hydrophobic nature of Mn_3_O_4_ (**Figure S3A**) 24, 25. To enable its use in biomedical applications, PAA was coated on the surface of UM to form the nanocomposite of UMP. As shown in **Figure 1B**, the modification of PAA did not alter the structure of UM. Energy-dispersive X-ray spectroscopy (EDS) elemental mapping in **Figure 1C** further confirmed that the core of UMP was made of UiO (Hf element) and the shell was made of Mn_3_O_4_ (Mn element). Additionally, the element line mapping and EDS analysis also gives the compositional and structural details, indicating Mn_3_O_4_ has been successfully coated onto UiO to form the core-shell structure (**Figure S4**). Besides, the prepared UMP displayed superior stability in water, PBS, and DMEM, even for one year (**Figure S3B-C**). There was no hemolysis to erythrocytes, which ensured the possibility of further application of UMP *in vivo* (**Figure S5**).

The successful synthesis of UiO, UM, and UMP was further confirmed by the powder X-ray diffraction (PXRD) analysis. As shown in **Figure 1E**, the diffraction peaks of UM matched reasonably well with the standard pattern of UiO and Mn_3_O_4_, indicating successful synthesis of UM. In addition, the coating of PAA had a negligible impact on the characteristic PXRD peaks and the UV-Vis spectra of UM (**Figure 1F**). The surface atomic composition of UM was determined by XPS. Since the inorganic shell of UM was comprised of Mn_3_O_4_, the two peaks of Mn 2p_1/2_ and Mn 2p_3/2_ at 652.8 and 641.1 eV with a spin energy separation of *ca* 11.7 eV were attributed to Mn_3_O_4_ (**Figure S6**) 26, 27. Compared with UiO, the absence of an N 1s peak and the presence of Mn 2p peaks further demonstrated that Mn_3_O_4_ forms a shell on UiO, and thus successful synthesis of UM.

Due to the presence of -NH_2_ group, UiO has a positively charged surface potential of +38.5 mV, while UM has a weak positive charge (+1.0 mV) due to the presence of negatively charged Mn_3_O_4_ (-12 mV) (**Figure 1G**). After electronegative PAA conjugation, the zeta potential reversed from a positive value of UM to a negative one of -16 mV for UMP, which confirmed successful modification of PAA on UM.

### UMP-mediated multiple strategies

**Figure 2:**
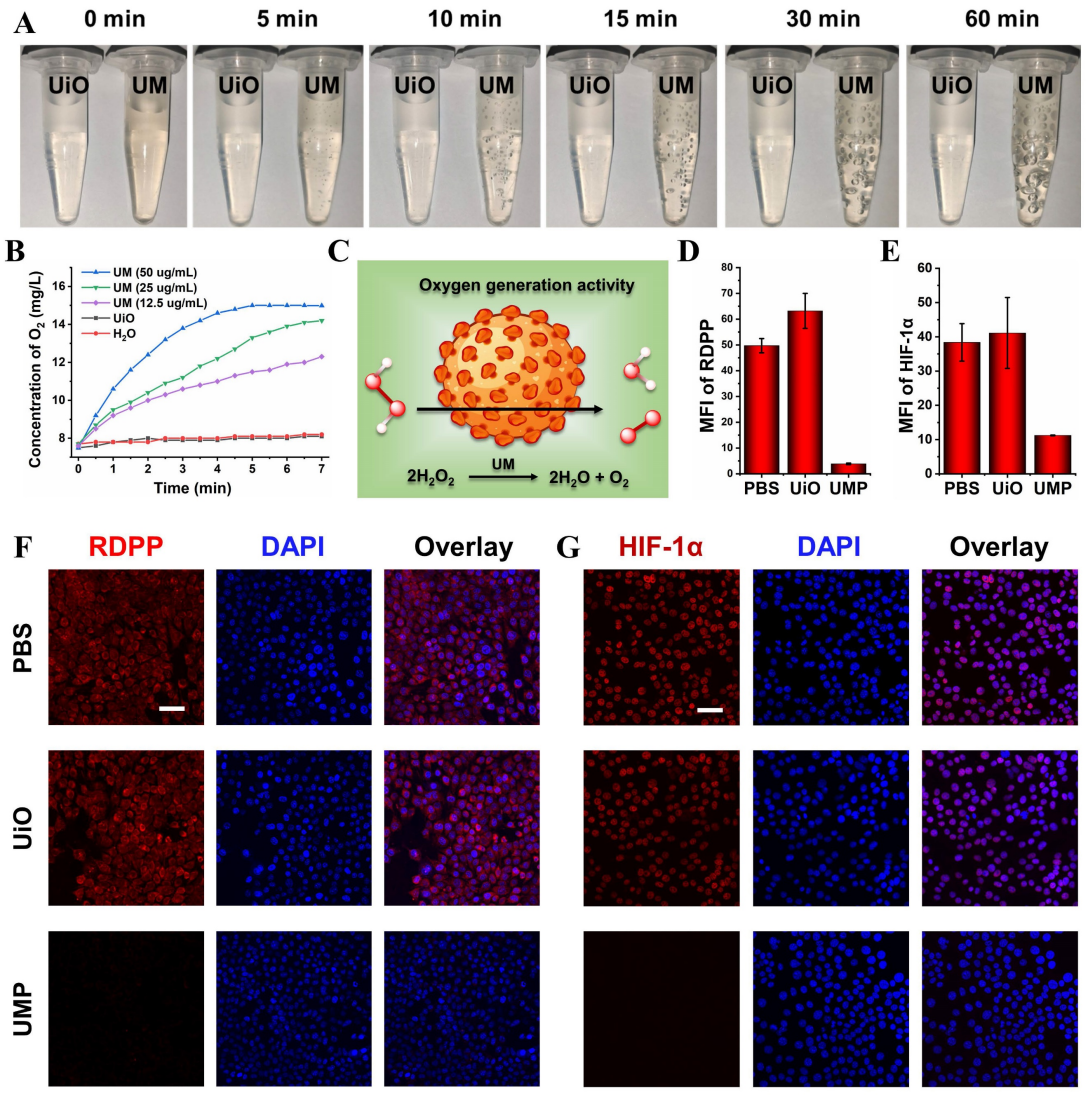
(A) The photos of UiO (100 μg/mL) and UM (50 μg/mL) in 10 mM H_2_O_2_ solution showing the generation of O_2_ bubbles. (B) The oxygen generation profiles of H_2_O, UiO (100 μg/mL), and UM (12.5, 25, 50 μg/mL) in 1 mM H_2_O_2_ solution. (C) Schematic illustration of the oxygen generation activity of UM. The quantitative analysis of the the mean fluorescence intensity (MFI) of RDPP (D) and HIF-1α (E) (data are presented as means ± SD; n = 3). (F) The CLSM images of oxygen generation in 4T1 cells with different treatments under the hypoxia condition and stained with RDPP as a hypoxia probe (Ex: 488 nm, scale bar = 50 μm). (G) The CLSM images of HIF-1α expression in 4T1 cells with different treatments under the hypoxia condition (scale bar = 50 μm).

Obvious bubbles could be observed after incubating UM with 10 mM H_2_O_2_ while mixing UiO and H_2_O_2_ did not generate such bubbles (**Figure 2A**). A dissolved oxygen analyzer (JPB-607A) was used to identify that the produced bubbles were oxygen. As shown in **Figure 2B**, the real-time oxygen concentration increased rapidly after the addition of UM into the H_2_O_2_ solution, while UiO and the control group exhibited almost no oxygen-generation ability. These results showed that the oxygen generation was derived from the reaction between Mn_3_O_4_ and H_2_O_2_ (**Figure 2C**).

4T1 cells were employed to evaluate the intracellular oxygen generation effects of UMP. As shown in **Figure S7**, UMP could be efficiently uptaken by 4T1 cells. We used cell-permeable [Ru(dpp)_3_]Cl_2_ (denoted as RDPP, dpp = 4,7-diphenyl-1,10-phenanthroline), which is a hypoxia indicator with red phosphorescence at 615 nm, to confirm the capability of alleviating hypoxia of UMP. As depicted in **Figure 2D** and **Figure 2F**, 4T1 cells exhibited intense red fluorescence under hypoxic conditions when treated with PBS or UiO. When 4T1 cells were treated with UMP, RDPP displayed weak red fluorescence due to the abundant O_2_ generated by UMP. The reversal of hypoxia by UMP in 4T1 cells was further demonstrated by HIF-1α staining, which is an intranuclear protein and upregulated in cells at low O_2_ concentrations. As shown in **Figure 2E** and **Figure 2G**, PBS and UiO-treated 4T1 cells showed high HIF-1α signals, while UMP-treated 4T1 cells showed negligible HIF-1α signals because of UMP-mediated oxygen generation.

**Figure 3:**
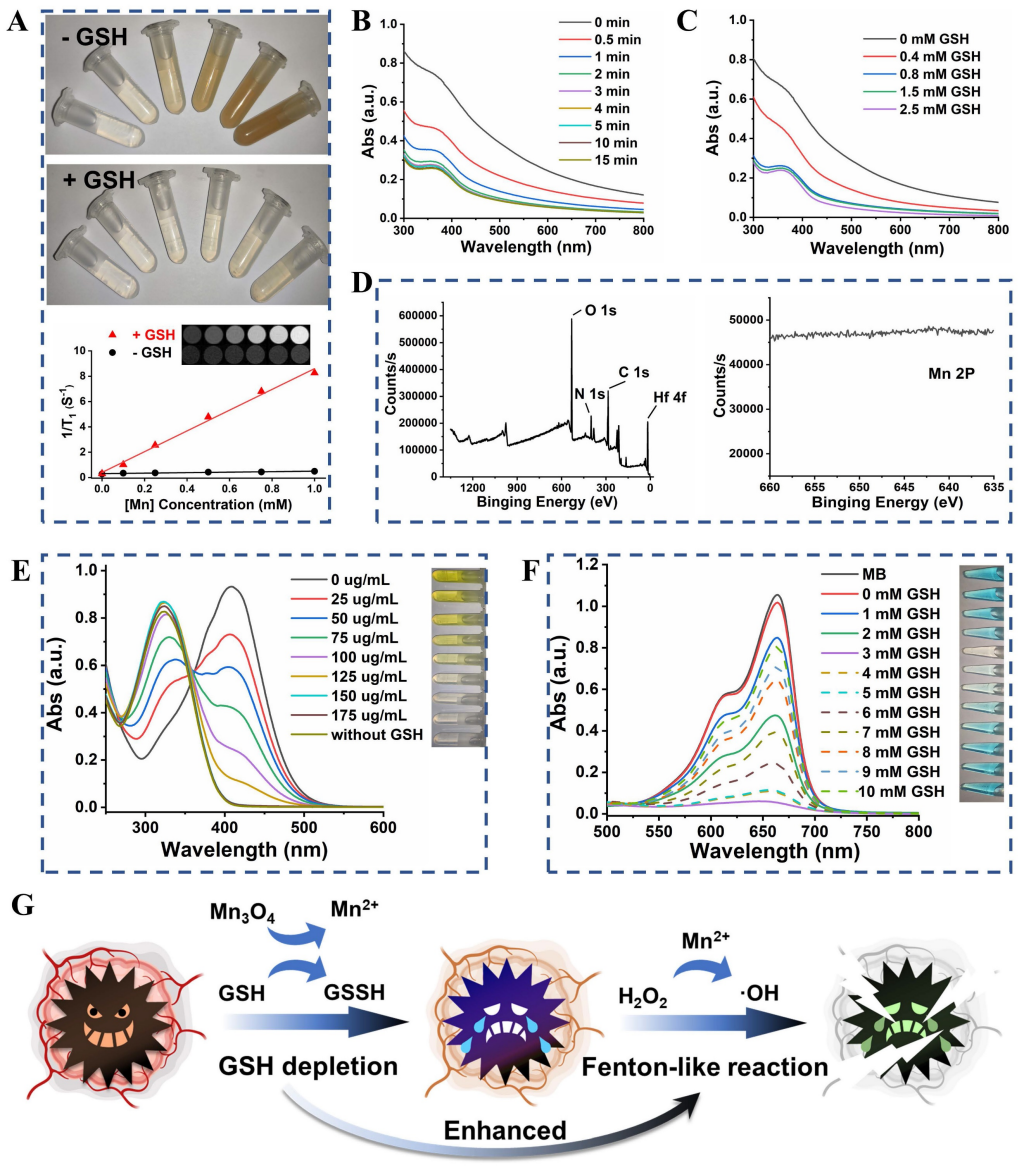
(A) The photos and T_1_-weighted MRI of UM solution at different Mn concentrations (0, 0.1, 0.25, 0.5, 0.75, and 1 mM) with or without 10 mM GSH treatment. (B) The UV-Vis absorption spectra of UM (50 μg/mL) when treated with 1 mM GSH at different times. (C) The UV-Vis absorption spectra of UM (50 μg/mL) when treated with different concentrations of GSH for 15 min. (D) The full and Mn 2p XPS spectra of UM when treated with 10 mM GSH. (E) The UV-Vis absorption spectra of DTNB after degradation by GSH-treated with different concentrations of UM. Inset: the corresponding photo. (F) The UV-Vis absorption spectra of MB after degradation by H_2_O_2_ plus GSH-treated UM. Inset: the corresponding photo. (G) Schematic illustration of GSH depletion enhanced Fenton-like reaction.

Color changes (from brown to colorless) of UM were observed after treatment with different concentrations of GSH (**Figure S8**), which provided initial evidence that the UM could react with GSH. The Mn^2+^ released from UM showed GSH concentration dependency (**Figure S9**) and enabled T_1_-weighted magnetic resonance imaging (MRI) of different concentrations of UM solution 28-30. As shown in **Figure 3A**, UM treated with GSH exhibited a paramagnetic property with an r_1_ value of 8.20 mM^-1^ s^-1^ and the T_1_-field MRI signal gradually increased with increased UM concentration. Unsurprisingly, UM in the absence of GSH had a weak T_1_-field MRI signal with an r_1_ value of 0.19 mM^-1^ s^-1^, highlighting that UM could be used for GSH-activated MRI *in vivo*.

The UV-Vis absorption spectra suggested that with the increase of GSH concentration from 0 to 2.5 mM, the absorbance of UM gradually decreased, further proving that Mn_3_O_4_ on UM was decomposed by GSH at 15 min (**Figure 3B**-**C**). The presence of PAA did not prevent the decomposition of UM by GSH (**Figure S10**). After the Mn_3_O_4_ surface was peeled by the GSH solution, it can be seen from the XPS results that the Mn 2p peaks disappeared and the N 1s peak appeared, which further proved that Mn_3_O_4_ was degraded by GSH (**Figure 3D**).

The GSH depletion was verified using 5,5'-dithiobis-(2-nitrobenzoic acid) (DTNB) based on its specificity to be reduced by GSH (**Figure S11**) to form 5-thio-2-nitrobenzoic acid (TNB) with the adsorption peak at 412 nm. As shown in **Figure 3E**, as the concentration of UM increased, the yellow color of TNB gradually weakened until disappeared, indicating that GSH was completely consumed by UM. The Mn^2+^-mediated Fenton-like reaction was further explored (**Figure 3F**). The •OH-induced methylene blue (MB) oxidation absorption (*ca* 660 nm, from blue to colorless) notably declined with the increment of GSH concentrations (0 to 3 mM) compared with that of the GSH-free group, implying more •OH production by GSH-mediated Mn^2+^ release. However, the MB degradation weakened when GSH increased from 4 mM to 10 mM, due to the ROS scavenging by excessive GSH. The •OH generation was also investigated by the EPR spectroscopy (**Figure S12**). After adding H_2_O_2_, the strong EPR signal in 3 mM GSH-pretreated UM group demonstrated the •OH generation, while there was no •OH generation in the other groups.

Given the excellent GSH consumption and •OH production capacity of UM, we speculated that UM will undergo a redox reaction with GSH in the cells to generate Mn^2+^ with Fenton-like activity, thereby converting endogenous H_2_O_2_ into highly toxic •OH. The consumption of the antioxidant GSH in the cell prevents the elimination of •OH, leading to the enhancement of Fenton-like reaction (**Figure 3G**).

### Intracellular therapeutic effects

**Figure 4:**
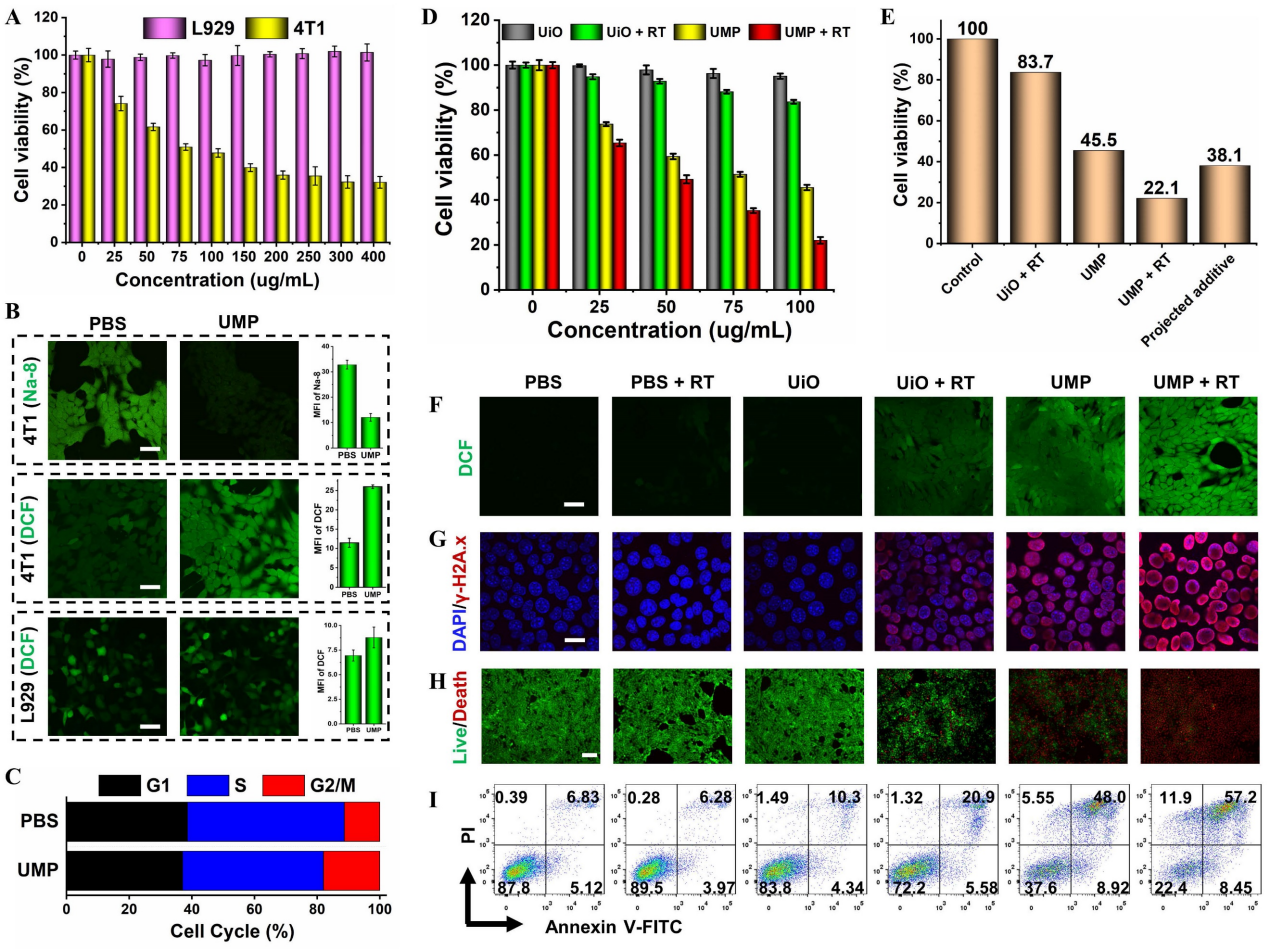
(A) The cell viability of 4T1 and L929 cells treated with different concentrations of UMP for 24 h. (B) The intracellular GSH (Na-8) and ROS (DCFH-DA) levels after being treated with PBS or UMP (scale bar = 50 μm). The quantitative analysis of MFI of Na-8 and DCF. (C) The re-assortment of the cell cycle with the treatment of PBS or UMP. (D) The cell viability of 4T1 cells treated with different concentrations of UiO and UMP with or without X-ray irradiation. (E) The cell viability of 4T1 cells treated with the same concentration (100 μg/mL). The projected additive value was calculated by multiplying the cell viability of the UiO + RT and UMP groups 31. (F) The CLSM images of 4T1 cells with different treatments and stained with DCFH-DA (scale bar = 50 μm). (G) The immunofluorescent imaging of γ-H2A.x in 4T1 cells with different treatments (scale bar = 20 μm). (H) The live/dead staining assay with various treatments using Calcein-AM/PI kit (scale bar = 100 μm). (I) The apoptosis analysis after different treatments. Data are represented as means ± SD; n = 3.

Encouraged by the excellent GSH elimination and •OH generation capacity *in vitro*, the cytotoxic effect of UMP was compared in the normal L929 and cancerous 4T1 or MCF-7 cell lines (**Figure 4A** and** S13**). As expected, UMP had a negligible effect on L929 cells due to the low levels of GSH and H_2_O_2_.

In contrast, UMP significantly decreased the percentage of viable 4T1 or MCF-7 cells. This could be attributed to the high GSH content of tumor cells that promote the degradation of UMP, leading to the Mn^2+^-mediated Fenton-like reaction and thereby inducing redox imbalance in cancerous cells.

To test our hypothesis, we conducted a series of validation experiments at the cellular level in 4T1 cells. As shown in **Figure 4B**, the fluorescence level marginally decreased by 63.4% after incubation with UMP using a GSH detection probe (Na-8) 32, indicating intracellular GSH depletion. Besides, GSH concentration reduction upon UMP treatments at various concentrations has been further verified *via* DTNB assay with the blank group as control (**Figure S14**). The overexpressed GSH in cancerous cells serves to scavenge ROS to maintain their survival, depleting GSH should enhance the ROS accumulation. The intracellular ROS generation was monitored using the non-fluorescent DCFH-DA which can convert to the highly fluorescent DCF (green) in the presence of ROS 33. The treatment of 4T1 cells with UMP (50 μg/mL) showed a bright green fluorescence, indicative of efficient ROS production. In contrast, no obvious green fluorescence change was observed in L929 cells after UMP treatment as compared with the PBS group, further indicating that UMP is specific to cancer cells.

Studies have shown that ROS can arrest cell cycle at the G2/M phase 34-36. To investigate the effect of UMP on cell cycle distribution, 4T1 cells treated with UMP were subject to flow cytometric assay. The proportion of cells at the G2/M phases was increased in the UMP group (17.93%) compared to the PBS group (11.27%) (**Figure 4C**). Since the G2/M phases exhibit high sensitivity to radiation 37, 38, ROS induced by UMP could increase cellular radiosensitivity by regulating the cell cycle when implementing RT. Therefore, in combination with RT, the cell survival fraction decreased dramatically from 83.7% of UiO to 22.1% of UMP at a concentration of 100 μg/mL (**Figure 4D**), even 16.0% lower than the projected additive value (**Figure 4E**), implying that the sensitivity of 4T1 cells to X-ray was greatly enhanced after UMP pretreatment. As shown in **Figure 4F-I**, the UMP + RT group had the strongest ROS level and further induced the strongest DNA damage level, ultimately producing the strongest cell death effect. These results all indicated that UMP-mediated multiple strategies highly promote RT.

### ICD induction and TAMs reprogramming

**Figure 5:**
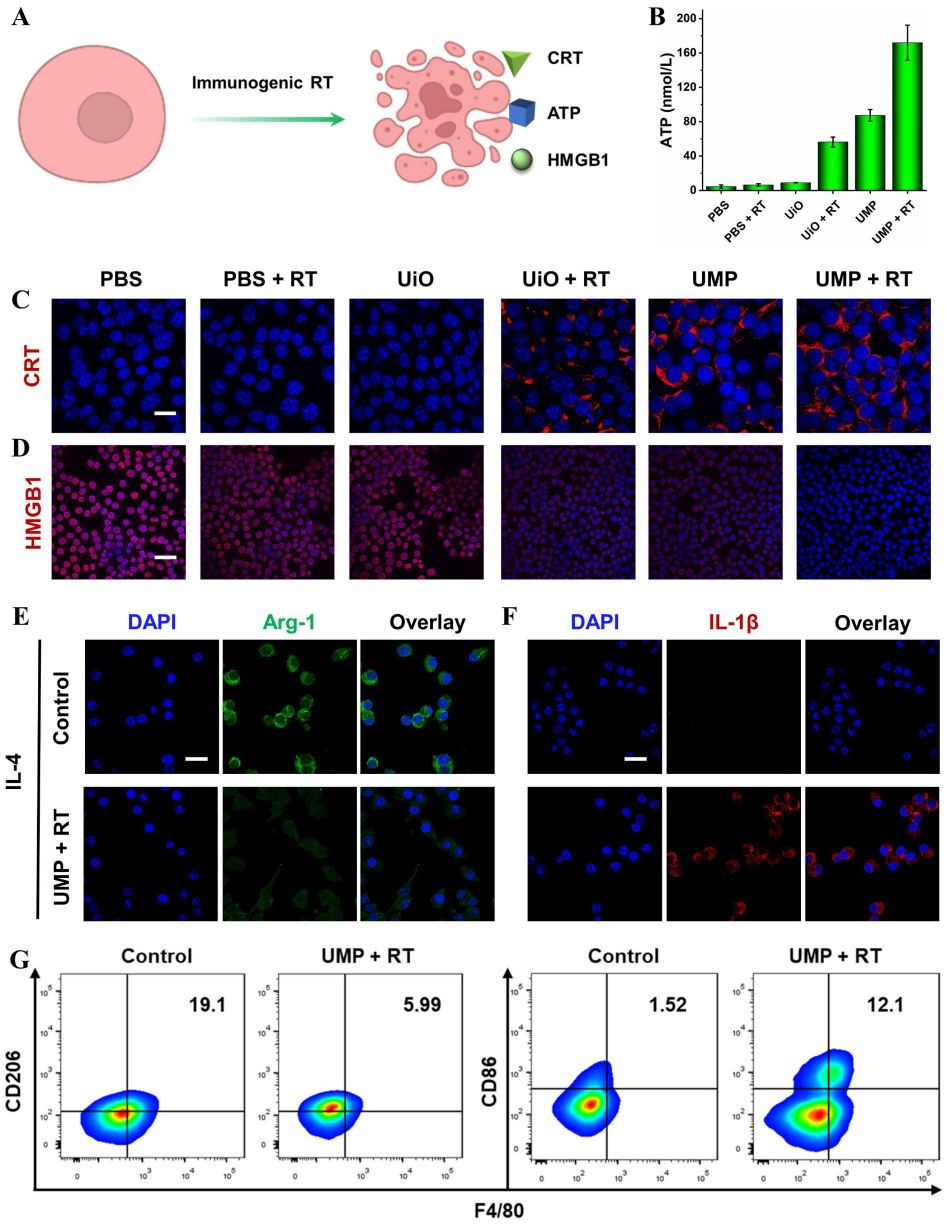
(A) Schematic representation of ICD induction *via* immunogenic RT. (B) The release of ATP in the culture medium with different treatments (data are represented as means ± SD; n = 3). The immunofluorescent imaging of CRT (C) and HMGB1 (D) in 4T1 cells with different treatments (scale bar = 20 μm for CRT, 50 μm for HMGB1). The immunofluorescent imaging of Arg-1 (E) and IL-1β (F) in IL-4-stimulated RAW 264.7 cells after incubation with UMP + RT-treated 4T1 cells culture medium (scale bar = 20 μm). (G) Flow cytometric analysis of CD86 and CD206 expression in IL-4-stimulated RAW 264.7 cells after incubation with UMP + RT-treated 4T1 cells culture medium

Immunogenic RT was evaluated by adenosine triphosphate (ATP) secretion, calreticulin (CRT) exposure and high mobility group box 1 (HMGB1) release (**Figure 5A**). As shown in **Figure 5B**, ATP levels in the supernatants showed a significant increase following the treatment of cells with UMP + RT. **Figure 5C** revealed that UMP + RT group displayed a dramatic increase in surface-exposed CRT, while PBS, PBS + RT, and UiO groups exhibited negligible CRT exposure. Moreover, 4T1 cells treated with UMP also presented substantial CRT exposure, indicating the intrinsic ability of UMP in inducing CRT translocation. When the cell is necrotic or damaged, HMGB1, a conserved nuclear protein, can be released to the outside of the cell. As indicated in **Figure 5D**, compared with the cells in the PBS group, 4T1 cells treated with UiO + RT or UMP exhibited weaker red fluorescence, and negligible fluorescence was observed in the UMP + RT group. This indicated that UMP + RT treatment significantly enhanced the release of HMGB1 from 4T1 cells.

As the major tumor-infiltrating immunosuppressive cells in the TME, TAMs are usually educated to present an M2 state, which always leads to immune evasion. Reversing the immunosuppressive TME *via* TAMs reprogramming has emerged as an effective therapeutic tactic for anti-tumor immunotherapy 17, 39. To verify the TAMs reprogramming, we pretreated RAW 264.7 cells with interleukin 4 (IL-4) for 24 h to generate M2 macrophages, then incubated them with UMP + RT-treated 4T1 cells culture medium. The immunofluorescence of arginase-1 (Arg-1, secreted by M2 macrophages) decreased while interleukin-1β (IL-1β, a proinflammatory factor secreted by M1 macrophages) increased in the UMP + RT treatment group (**Figure 5E**-**F**). The result of flow cytometric analysis similarly demonstrated a decrease in M2 macrophages (CD206) and an increase in M1 macrophages (CD86) in the UMP + RT group (**Figure 5G**).

### *In vivo* anti-tumor studies

**Figure 6:**
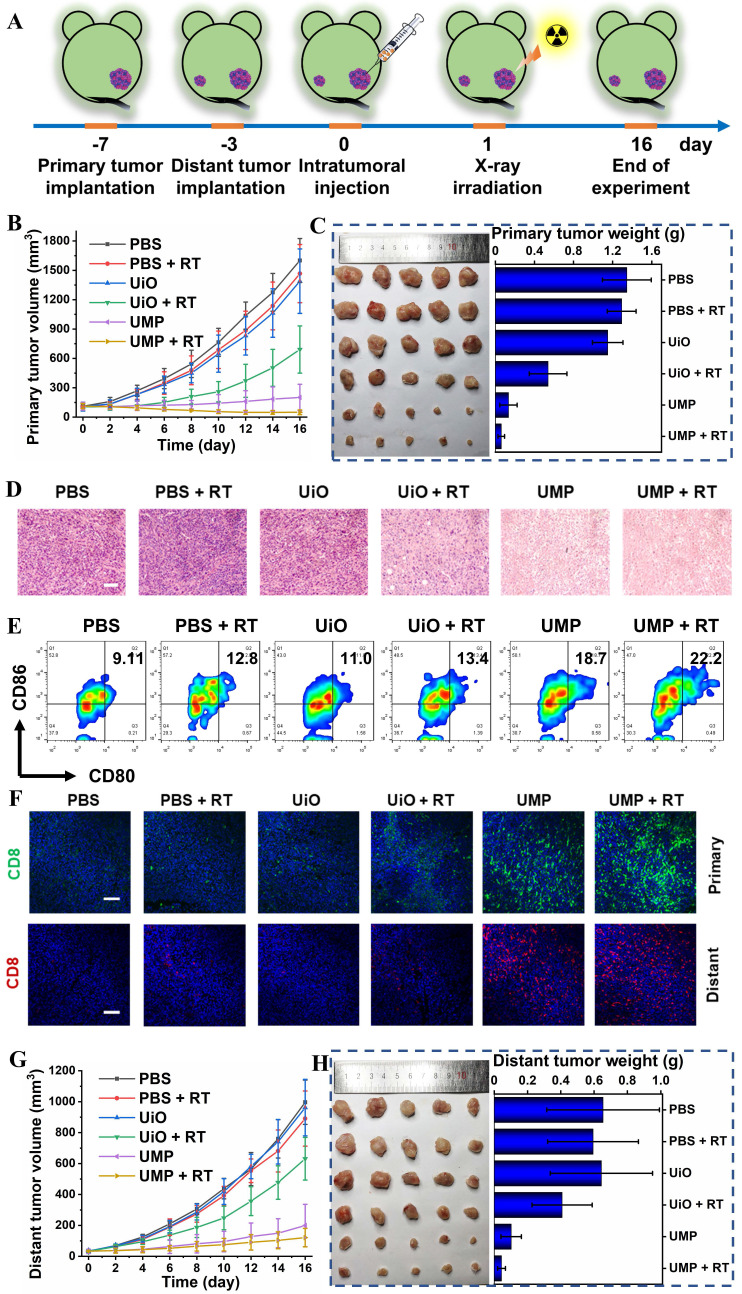
(A) Schematic illustration of the experimental design with bilateral 4T1 tumor-bearing BALB/c mice. (B) The primary tumor growth curves in different groups. (C) The primary tumor images and weight in different groups were obtained on the 16^th^ day. (D) The H&E staining of primary tumor sections from different groups (scale bar = 100 μm). (E) Representative flow cytometry plots of matured DCs (CD80^+^CD86^+^ gated on CD11c^+^ DCs) in the tumor-draining lymph nodes of 4T1 tumor-bearing mice. (F) The representative immunofluorescence staining images for CD8^+^ T cells of primary and distant tumor sections from different groups (scale bar = 100 μm, blue represents DAPI). (G) The distant tumor growth curves in different groups. (H) The distant tumor images and weight in different groups were obtained on the 16^th^ day. Data are represented as means ± SD; n = 5.

To verify the GSH-activated MRI of UMP at the tumor site, a bilateral BALB/c mouse model was established. As expected, an enhanced T_1_-weighted MRI signal intensity at the right tumor sites was observed after 24 h of intratumoral injection of UMP (**Figure S15**). Then a series of therapeutic experiments *in vivo* were conducted. As shown in **Figure 6A**, an anti-tumor study was performed in bilateral 4T1 breast tumor-bearing BALB/c mice *via* intratumoral injection in the primary tumor. Tumor growth inhibition (TGI) was calculated as (1 - mean weight of treated tumors/mean weight of control tumors) × 100%. The primary tumor growth curves in **Figure 6B** showed that PBS, PBS + RT and UiO treatment displayed negligible influence on the tumor progression. The UiO + RT treated group showed moderate tumor growth inhibition with a TGI of 37.5%, which was likely due to the radiosensitization effect of elemental Hf in UiO (**Figure 6C**). The growth of tumors in the UMP-injected group was inhibited by 90.0%, indicating that UMP-mediated ROS generation *via* GSH depletion-enhanced Fenton-like reaction could extremely inhibit the tumors. Significantly, when combined with 4 Gy radiation, UMP effectively regressed tumors to afford an impressive TGI of 95.5%. Besides, the results of hematoxylin and eosin (H&E) staining of tumor tissue slices also revealed that the tumors in groups UMP and UMP + RT showed much more severe cell apoptosis than those in the other groups (**Figure 6D**).

To clarify how the therapeutic benefits were induced by UMP, the TME modulation and anti-tumor immune responses were investigated. Tumor hypoxia is established as an important cause of radioresistance in solid tumors, and even predisposes the tumor to proliferation and metastasis 40. Given the ability of UMP to relieve hypoxia *in vitro*, we first verified whether UMP could relieve tumor hypoxia. As shown in **Figure S16**, the expression level of HIF-1α in tumor tissues after UMP treatment was lower than the other groups, indicating that UMP-mediated oxygen generation reduced the HIF-1α level. Notably, the higher expression of HIF-1α was observed after treatment with RT, probably because X-ray exacerbate tumor hypoxia 10, 41, 42.

We next determined DNA damage of different treatments by detecting γ-H2A.x. The significantly higher red γ-H2A.x fluorescence was observed in the group treated with UMP + RT than that treated with UMP or UiO + RT, while no fluorescence was observed in the other groups (**Figure S17**). We further evaluated their ability to trigger CRT exposure in primary tumor cells. As shown in **Figure S18**, the PBS, PBS + RT, and UiO groups failed to trigger the CRT exposure (green fluorescence), and UiO + RT group showed a slight exposure level, while a dramatic increase of CRT exposure in both UMP and UMP + RT groups were observed, suggesting the potent ICD-inducing effect *in vivo*. Besides, HMGB1 showed the opposite level in different groups. No obvious green fluorescence was observed in the UMP and UMP + RT groups, while the other groups showed a bright fluorescence, indicative of the efficient HMGB1 release (**Figure S19**).

TAMs could influence the TME in various ways. We next detected the proportion of TAMs in TME after the synergetic treatment. Compared with other groups, an obvious decrease of Arg-1 (orange signal) was observed in the UMP and UMP + RT groups, while IL-1β (green signal) level was increased, indicating that the immunosuppressive TME has been reversed (**Figure S20**). Then the activation of immune responses was further measured in lymph nodes. The maturation of DCs was essential for T cell activation. In the tumor-draining lymph nodes (TDLNs), the place where mature DCs present antigens to T lymphocytes, CD80^+^CD86^+^ mature DCs were quantified by using flow cytometry analyses. As presented in **Figure 6E**, a distinct improvement ratio of DCs maturation (9.11% to 22.2%) in TDLNs was found in UMP + RT group. The activation of immune responses was further measured in the primary and distant tumors (**Figure 6F**). The immunofluorescence images showed that a large number of CD8^+^ T cells accumulated at the primary and distant tumor region of the UMP + RT group, compared with the other groups, which proved that the tumor-specific immunity was activated. In addition, the highest secretion levels of tumor necrosis factor-α (TNF-α) and interferon-γ (IFN-γ) were displayed in the UMP + RT treatment group, suggesting that the immune response was effectively activated (**Figure S21**). These results all indicate that the UMP-mediated multiple strategies could effectively reverse the immunosuppressive TME and elicit intense immune responses, as demonstrated by a 93.2% distant tumor growth inhibition of the UMP + RT group (**Figure 6G**-**H**).

Notably, the UMP group showed a surprising distant tumor inhibition effect with a TGI of 84.4%, indicating that UMP-mediated multiple strategies could highly inhibit the distant tumor *via* reversing the immunosuppressive TME and activating CD8^+^ T cells. GSH acts as a co-factor in glutathione peroxidase 4 (GPX4)-catalyzed lipid repair systems, and therefore UMP-mediated GSH depletion can inactivate GPX4 to induce ferroptosis 43, 44. In contrast to bright green fluorescence in control tumors, negligible fluorescence was detected in the tumors treated with UMP, implying UMP-mediated GSH depletion-enhanced Fenton-like reaction indeed inactivated GPX4 to boost ferroptosis (**Figure S22**). The LPO level could be used as a significant indicator of ferroptosis. C11-BODIPY^581/591^, an oxidation-sensitive and LPO-specific fluorescent probe, is capable of accumulating at the cell membrane. Upon oxidation, the emission peaks of C11-BODIPY^581/591^ at 510 nm (green) gradually increase and maintain the intrinsic lipophilicity, facilitating the membrane LPO detection. As shown in **Figure S23**, the treatment of cancer cells with UMP showed a bright green fluorescence, indicative of efficient LPO production. To gain a deeper insight, after the cancer cells were pre-incubated with ferroptosis inhibitors GSH and ferrostatin-1 (Fer-1, a peroxyl radical scavenger), the cells displayed lower LPO signals, indicating that the ferroptosis was blocked. Mitochondrial membrane potential was measured by staining cells with tetramethyl rhodamine ethyl ester (TMRE), a kind of cell-permeable, cationic, and nontoxic fluorescent dye that specifically stains live mitochondria. As shown in **Figure S24**, compared with PBS group, 4T1 cells treated with UMP exhibited weaker red fluorescence, indicating the depolarization of the mitochondria membrane, which is another indicator of ferroptosis.

Studies have revealed that ferroptosis contributed to anti-tumor immunity by recruiting CD8^+^ T cells, whereas the activated CD8^+^ T cells promoted tumor ferroptosis *via* enhanced ferroptosis-specific lipid peroxidation in tumor cells 45, 46.

This mutually reinforced ferroptosis and CD8^+^ T cell recruiting may further explain the strong anti-tumor effect of UMP in both primary and distant tumors. In addition, it was reported that Mn-insufficient mice had significantly enhanced tumor growth and metastasis while intratumoral Mn injection effectively inhibited tumor growth both in primary and distant tumors, similarly demonstrating great potential of Mn-based nanomaterials in the activation of systemic immune response 47.

Since UiO did not have the ability to reverse the immunosuppressive TME, the UiO + RT group showed a slight degree of distant tumor growth inhibition with a TGI of 37.6%. The distant tumor growth curves in **Figure 6G** showed that PBS, PBS + RT and UiO treatment displayed negligible influence on tumor growth. Finally, both similar trends in body weights (**Figure S25**) and normal histological images of major organ slices (**Figure S26**) showed that UMP-mediated multiple strategies for immunogenic RT were systemically non-toxic.

Based on the above results, we speculated that UMP-mediated multiple strategies could activate a systemic immune response to inhibit cancer metastasis (the most common cause of cancer death) 48, 49. The incidence of breast cancer metastasis was up to 50%, and the prognosis of patients was poor, with an overall 5-year survival rate of about 27% 50. As shown in **Figure S27**, there were fewer breast metastases in the UMP and UMP + RT groups, while obvious tumor metastases were found in other groups. H&E staining study further showed the strong growth inhibitory effects of UMP on lung metastases with a significant decrease in the number and size of visible metastatic nodules. This result revealed that UMP-based multiple strategies for immunogenic RT can not only destroy the primary tumor but also produce a predominant abscopal anti-tumor effect to suppress the tumor metastasis.

## Conclusion

In summary, we have demonstrated that UMP-based multiple strategies could serve as an effective modulator of immunogenic RT by reversing the immunosuppressive TME, triggering the ICD effect, reprogramming TAMs, and initiating anti-tumor immune responses. In this study, core-shell UMP was chosen as an oxygen generator, GSH depletor, cell cycle regulator, and radiosensitizer to orchestrate the workable ICD effect. More importantly, with the potent ICD induction and reprogrammed TAMs, these multiple strategies could effectively mature DCs and produce full activation of anti-tumor T-cell immunity, which observably enhanced therapeutic effects on primary and distal/metastatic tumors. Taken together, it is likely that immunogenic RT triggered by nanomaterials-mediated multiple strategies would offer a new strategy for future oncology therapeutics.

## Supplementary Material

Supplementary figures and table.

## Abbreviations

TME: tumor microenvironment
RT: radiation therapy
ICD: immunogenic cell death
GSH: glutathione
TAMs: tumor-associated macrophages
G2/M: gap 2/mitosis
TDLNs: tumor-draining lymph nodes
DCs: dendritic cells
DAPI: 4',6-diamidino-2-phenylindole
DCF: fluorescent 2',7'-dichlorfluorescein
RDPP: [Ru(dpp)_3_]Cl_2_
MB: methylene blue
DTNB: 5, 5′-dithiobis-(2-nitrobenzoic acid)
Fer-1: Ferrostatin-1
TMRE: tetramethylrhodamine ethyl ester
DMPO: 5, 5-dimethyl-l-pyrroline-N-oxide
EPR: Electron paramagnetic resonance
LPO: lipid peroxide
DLS: dynamic light scattering
ROS: reactive oxygen species
H_2_O_2_: Hydrogen Peroxide
TEM: transmission electron microscope
SEM: scanning electron microscope
EDS: energy-dispersive X-ray spectroscopy
PDI: polymer dispersity index
PXRD: powder X-ray diffraction
XPS: X-ray photoelectron spectroscopy
ICP-MS: inductively coupled plasma-mass spectrometry
MRI: magnetic resonance imaging
CLSM: confocal laser scanning microscope
HIF-1α: hypoxia inducible factors-1α
MTT: methylthiazoletatrezolium
PI: propidium iodide
ATP: adenosine triphosphate
CRT: calreticulin
HMGB1: high mobility group box 1
IL-4: interleukin 4
Arg-1: arginase-1
IL-1β: interleukin-1β
TNF-α: tumor necrosis factor-α
IFN-γ: interferon-gamma
TGI: tumor growth inhibition
GPX4: glutathione peroxidase 4
H & E: hematoxylin and eosin
SD: standard deviation
