# Supplementary material for: Immunogenic radiation therapy for enhanced anti-tumor immunity via core-shell nanocomposite-mediated multiple strategies
Source: Theranostics. 2023 Jul 14;13(12):4121–37. doi: 10.7150/thno.84500 (PMC10405846; doi:10.7150/thno.84500)
Supplement: Supplementary file 1 — Supplementary figures and table. [file thnov13p4121s1.pdf]

Supporting Information

Immunogenic radiation therapy for enhanced anti-tumor immunity *via* core-shell nanocomposite-mediated multiple strategies

Naihan Huang[1], Along Qian[1], Yiming Zou[1], Miaoli Lin[1], Weilun Pan[1], Ming Chen[1], Wei Meng[1], Wenhua Zhang[2], Jinxiang Chen[1,*]

1. Guangdong Provincial Key Laboratory of New Drug Screening, Guangzhou Key Laboratory of Drug Research for Emerging Virus Prevention and Treatment, NMPA Key Laboratory for Research and Evaluation of Drug Metabolism, School of Pharmaceutical Sciences, Southern Medical University, Guangzhou, Guangdong 510515, China.
2. College of Chemistry, Chemical Engineering and Materials Science, Soochow University, Suzhou 215123, China.

* Corresponding author

E-mails addresses: jxchen@smu.edu.cn (J. Chen)

Table S1. Size and PDI of UiO obtained from different ratios of water and acetic acid *via* DLS measurement.

Water (mL)	Acetic acid (mL)	Size (nm)	PDI
40	60	726	0.156
60	40	318	0.123
70	30	268	0.074
80	20	144	0.038
90	10	91	0.151

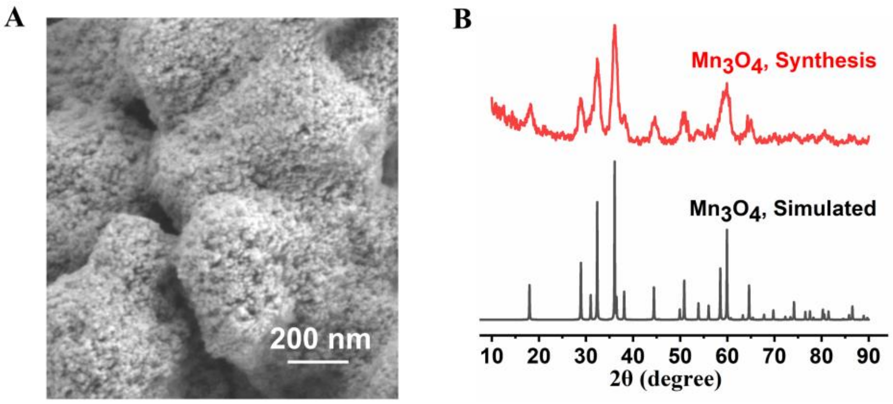

Figure S1. The SEM image (A) and PXRD (B) of Mn_3O_4.

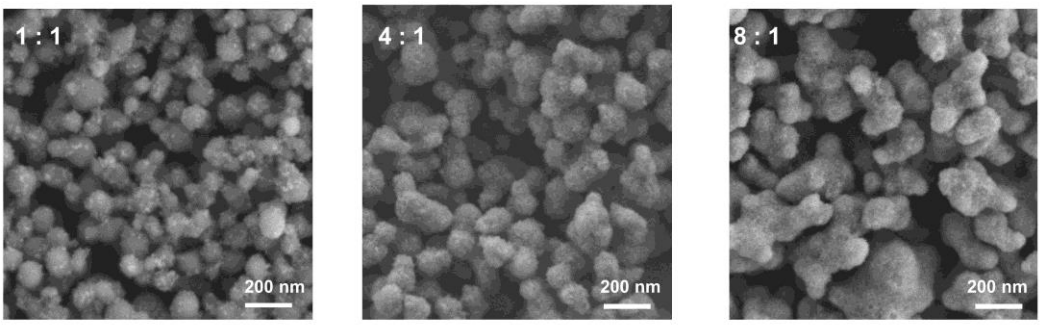

Figure S2. The SEM images of UM obtained from different mass ratios between $Mn(OAc)_2 \cdot 4H_2O$ and UiO.

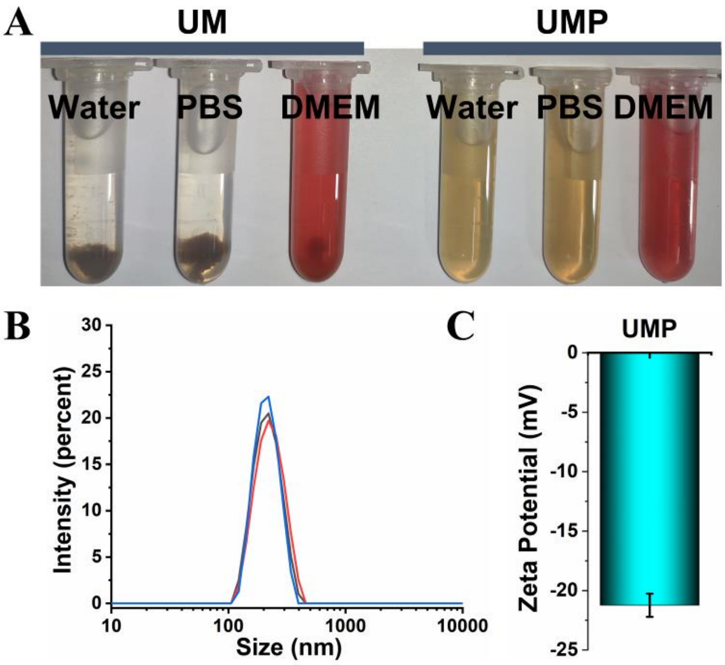

Figure S3. (A) The photo of UM and UMP in water, PBS, and DMEM for 24 h. The size distribution (B) and the zeta potential (C) of UMP in water for one year.

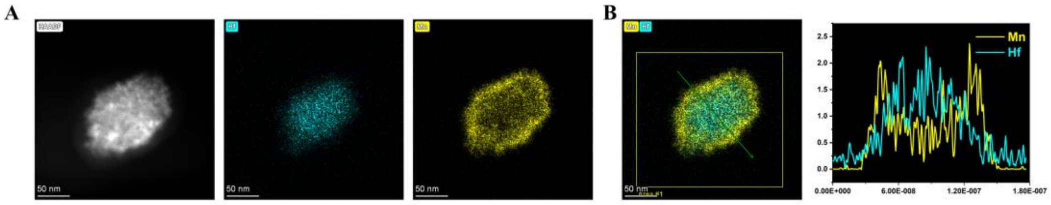

Figure S4. (A) The STEM-HAADF image and corresponding EDS elemental mapping of Hf and Mn of UM. (B) Element line scanning of UM.

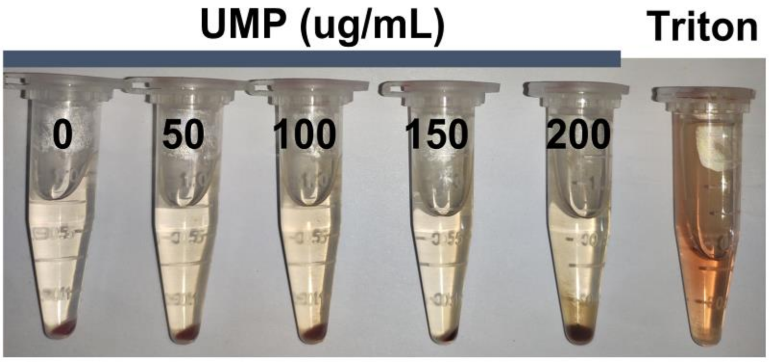

Figure S5. The photo showing the hemolytic activity of UMP at different concentrations (1% Triton X-100 as the positive control).

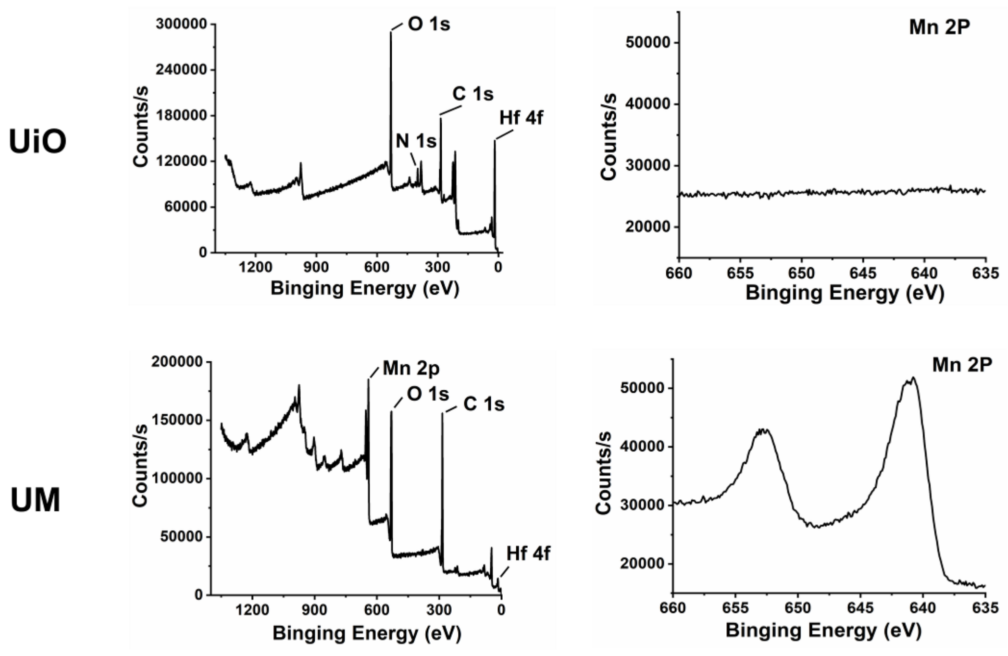

Figure S6. The full and Mn 2p XPS spectra of UiO and UM.

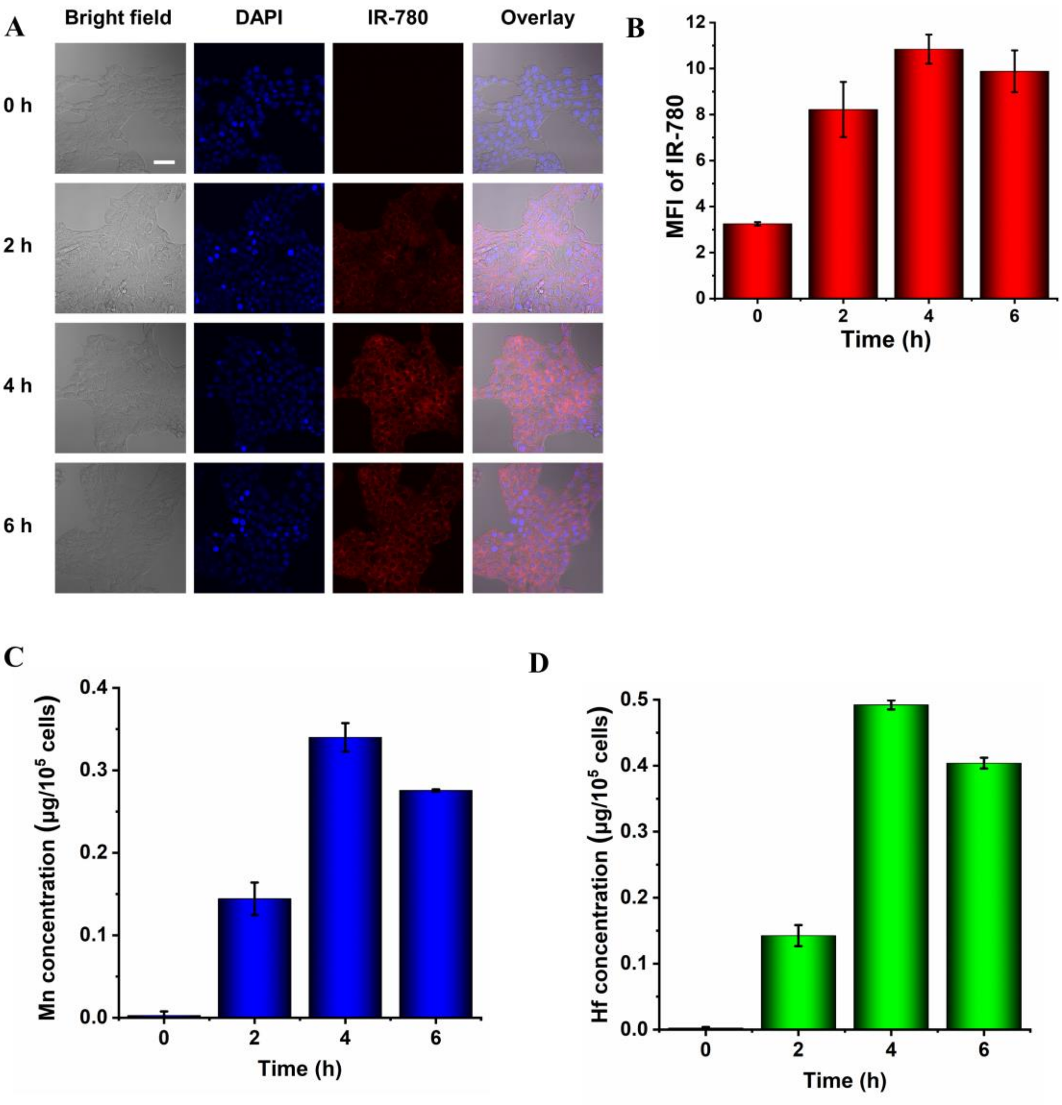

Figure S7. (A) The cellular uptake of PAA-coated UM@IR780 (50 μg/mL) at different times under CLSM observation (Ex: 640 nm, scale bar = 50 μm). (B) The quantitative analysis of the mean fluorescence intensity (MFI) of IR780 at different times. The Mn (C) and Hf (D) content of UMP-treated 4T1 cells at different times via ICP-MS. Data are presented as means ± SD; n = 3.

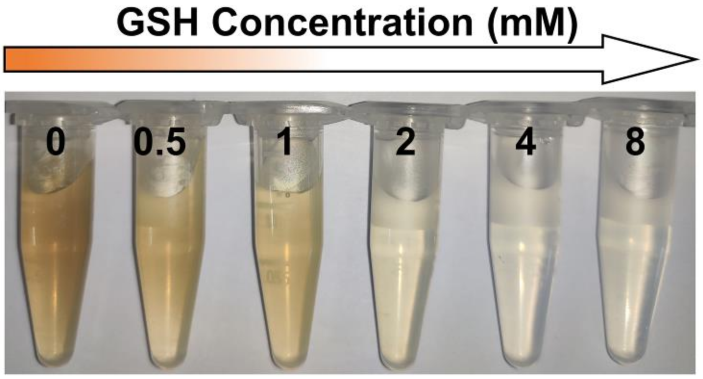

Figure S8. The photo of UM (200 μg/mL) when treated with different concentrations of GSH.

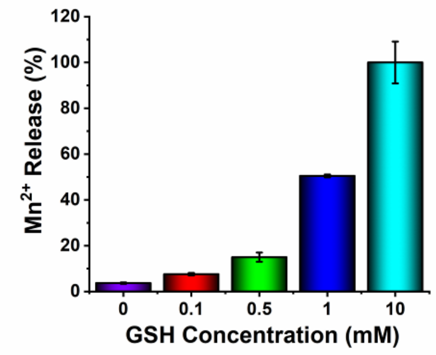

Figure S9. The Mn^{2+} release from UM (200 μg/mL) when treated with different concentrations of GSH.

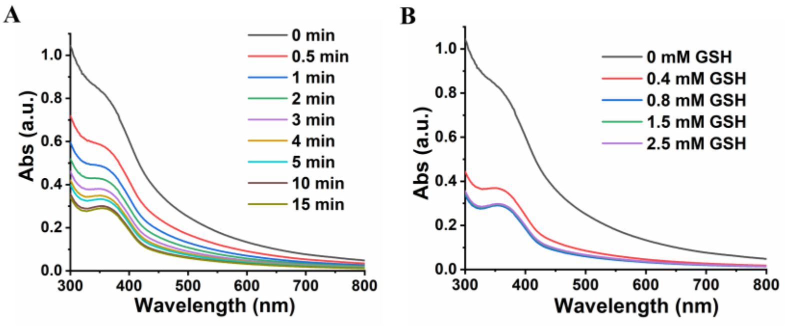

Figure S10. (A) The UV-Vis absorption spectra of UMP when treated with 1 mM GSH at different times. (B) The UV-Vis absorption spectra of UMP when treated with different concentrations of GSH for 15 min.

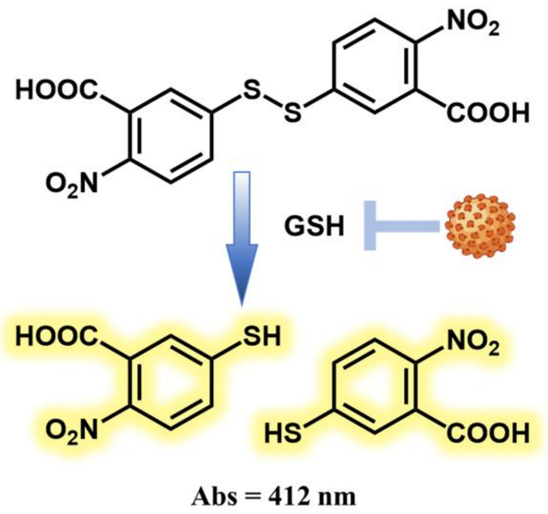

Figure S11. Schematic illustration of GSH detection *via* converting DTNB (up) to TNB (down).

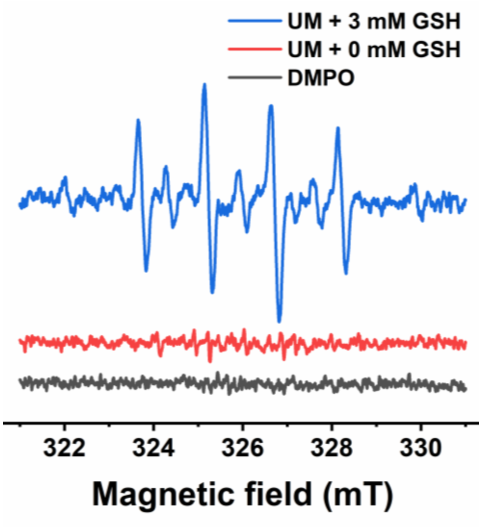

Figure S12. EPR spectra of different reaction systems with DMPO as the spin trap.

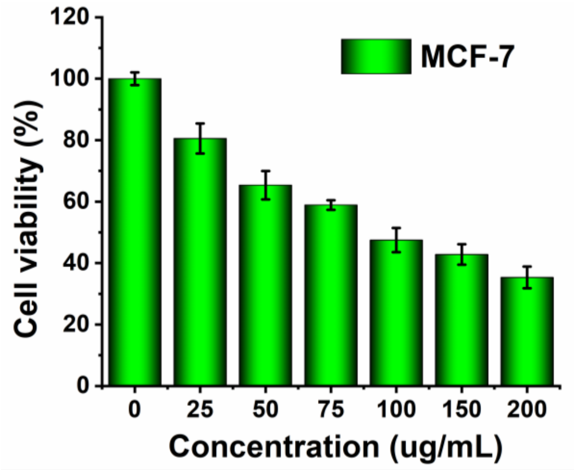

Figure S13. The cell viability of MCF-7 cells treated with different concentrations of UMP for 24 h. Data are represented as means ± SD; n = 3.

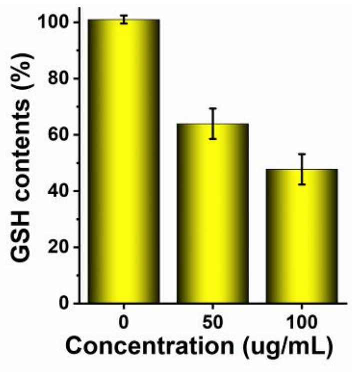

Figure S14. The GSH levels of 4T1 cells after incubation with different concentrations of UMP (data are presented as means ± SD; n = 3).

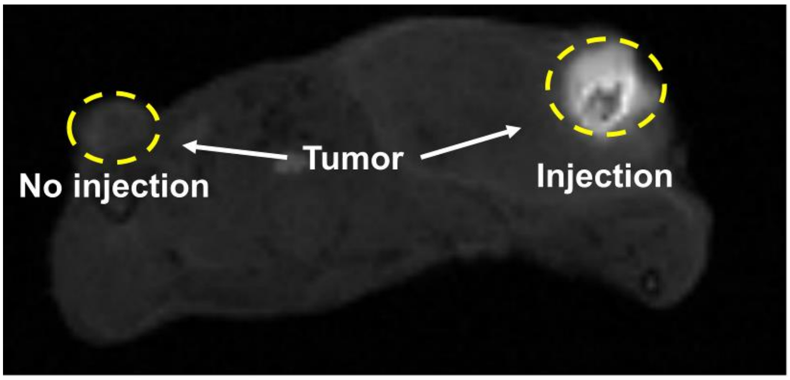

Figure S15. T_1-weighted MRI of a bilateral 4T1 breast tumor-bearing BALB/c mouse after intratumoral injection of UMP for 24 h.

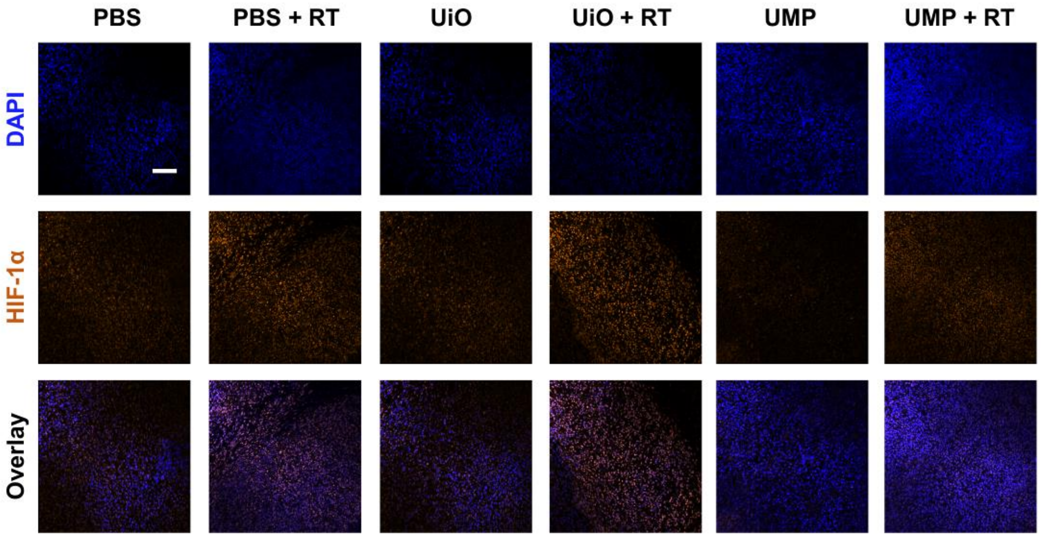

Figure S16. The representative immunofluorescence staining images for HIF-1α of primary tumor sections from different groups (scale bar = 100 μm).

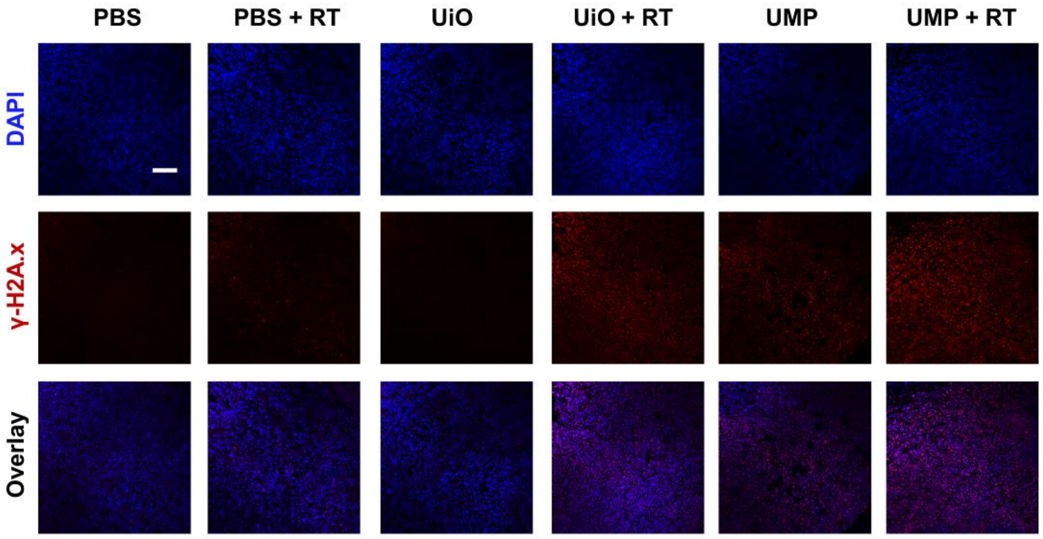

Figure S17. The representative immunofluorescence staining images for γ-H2A.x of primary tumor sections from different groups (scale bar = 100 μm).

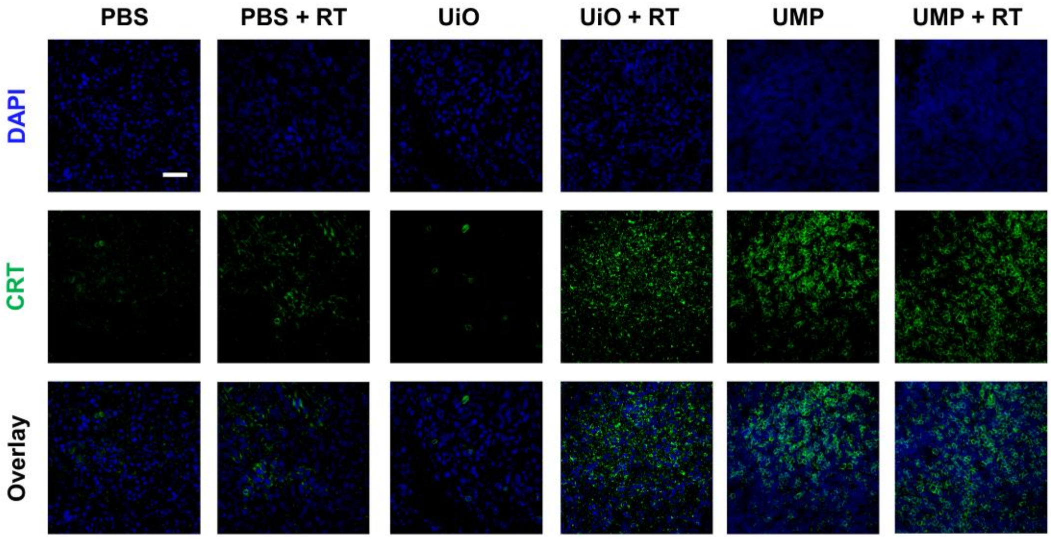

Figure S18. The representative immunofluorescence staining images for CRT of primary tumor sections from different groups (scale bar = 50 μm).

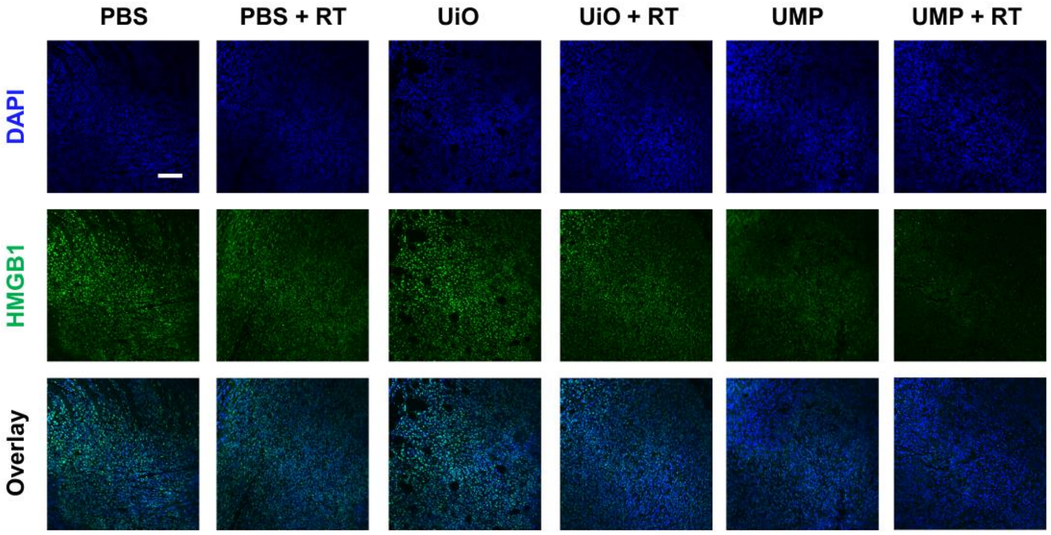

Figure S19. The representative immunofluorescence staining images for HMGB1 of primary tumor sections from different groups (scale bar = 100 μm).

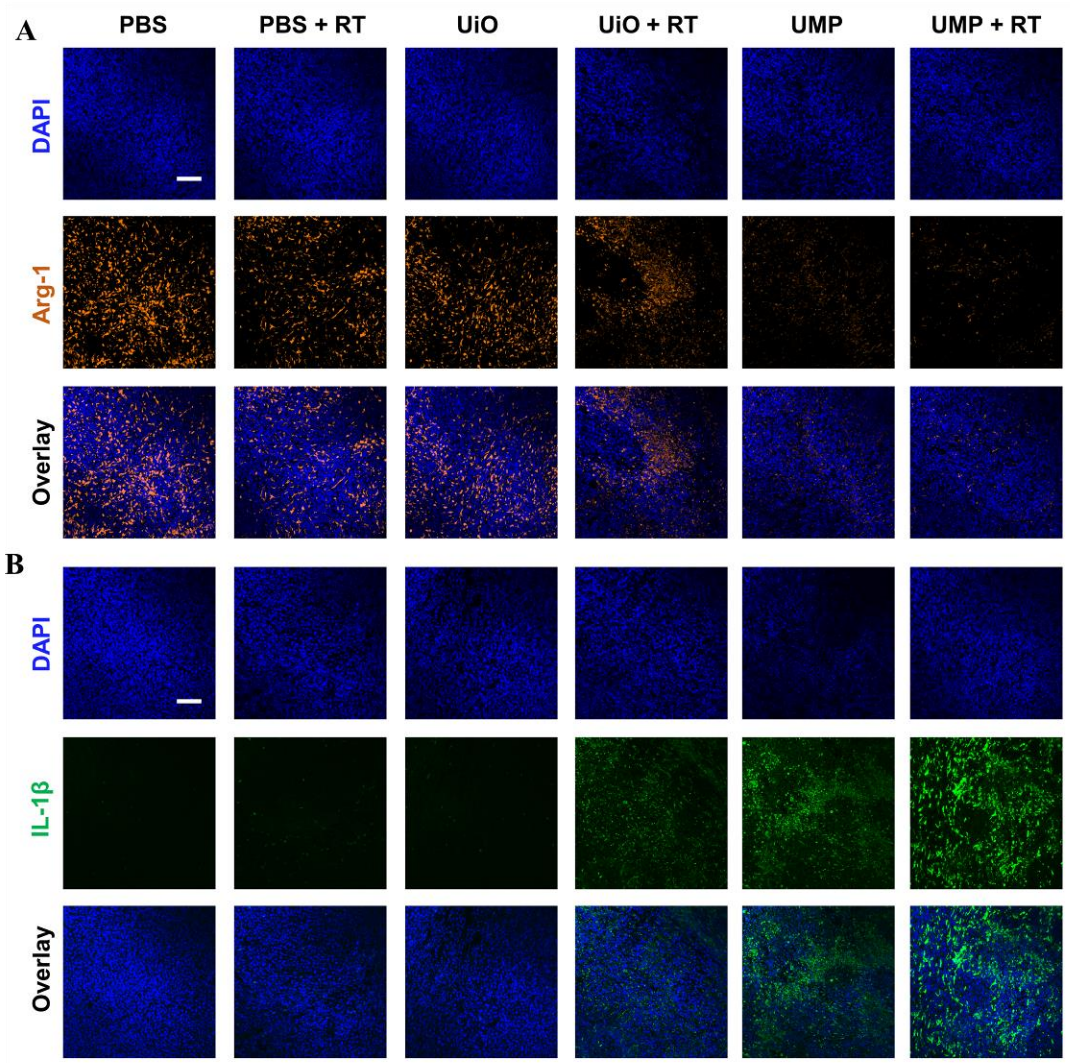

Figure S20. The representative immunofluorescence staining images for Arg-1 (A) and IL-1β (B) of primary tumor slices from different groups (scale bar = 100 μm).

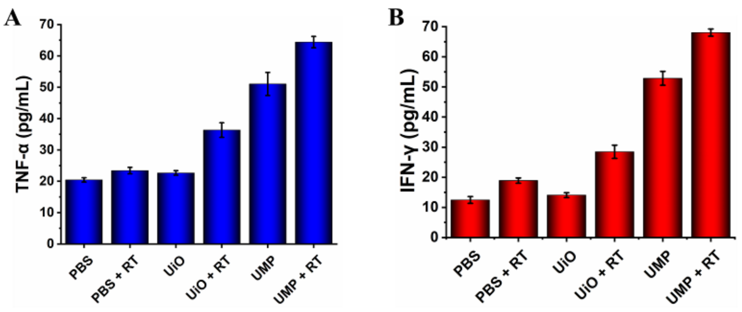

Figure S21. Cytokine levels of TNF-α (A) and IFN-γ (B) in sera from mice isolated post various treatments.

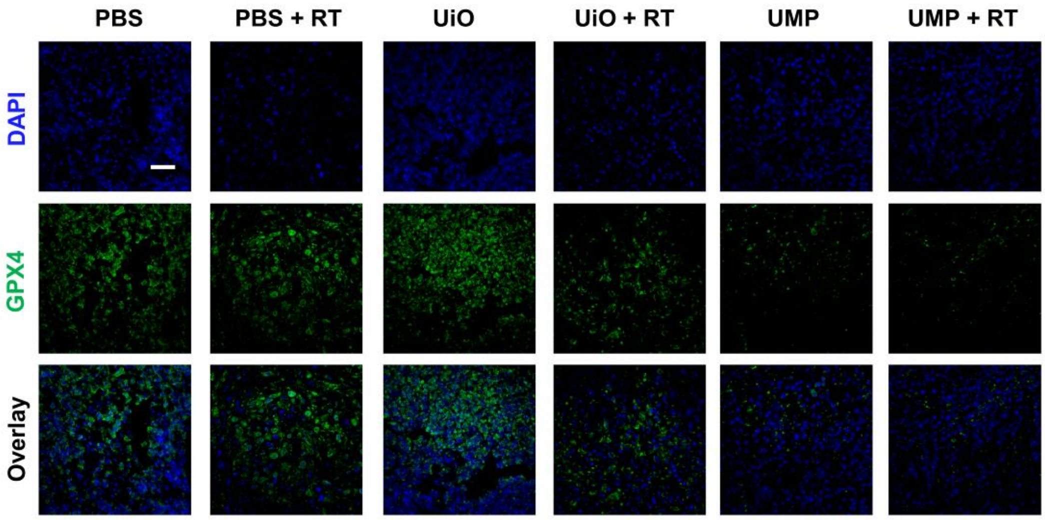

Figure S22. The representative immunofluorescence staining images for GPX4 of primary tumor sections from different groups (scale bar = 50 μm).

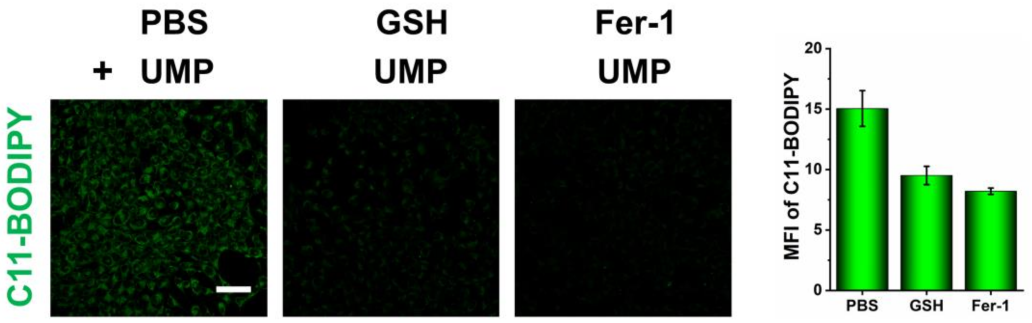

Figure S23. The CLSM images of 4T1 with different treatments and stained with C11-BODIPY$^{581/591}$ (scale bar = 50 μm). The quantitative analysis of the MFI of C11-BODIPY$^{581/591}$. Data are presented as means ± SD; n = 3.

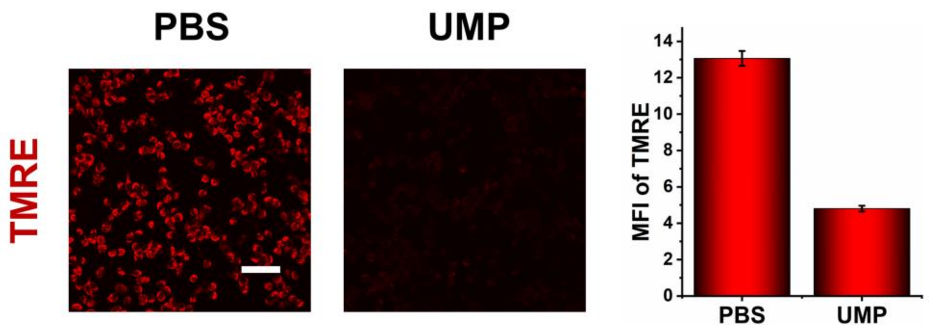

Figure S24. The CLSM images of 4T1 with different treatments and stained with TMRE (scale bar = 50 μm). The quantitative analysis of the MFI of TMRE (data are presented as means ± SD; n = 3).

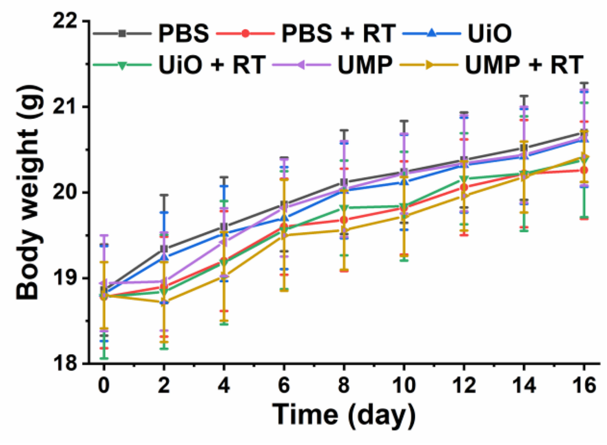

Figure S25. The body-weight changes in different groups.

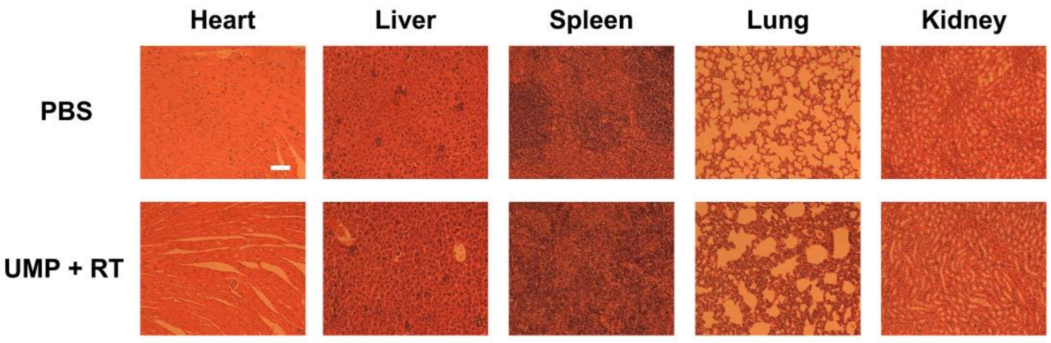

Figure S26. The H&E staining of main organs (heart, liver, spleen, lung, and kidney) tissue slices in PBS and UMP + RT groups (scale bar = 100 μm).

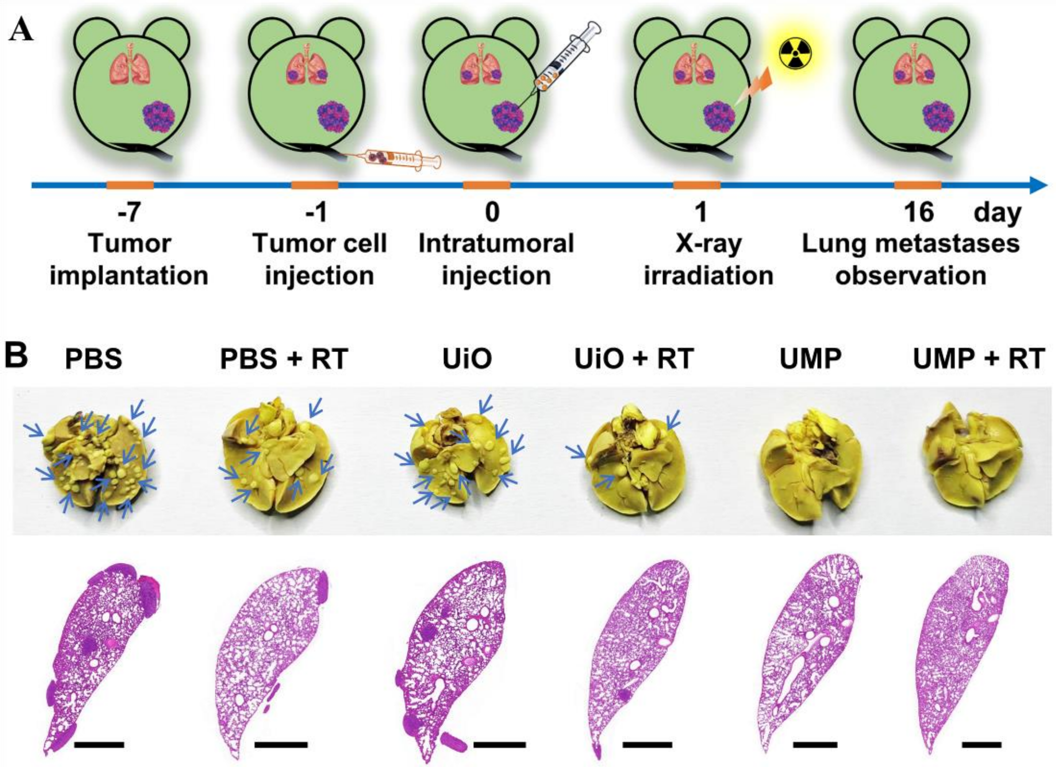

Figure S27. (A) Schematic illustration of the experimental design with lung metastatic BALB/c mice. (B) The images of lung fixed by Bouin's solution and H&E sections of lungs (the arrows indicate metastatic tumor nodules, scale bar = 1 mm).
